# Advanced Architectures and Relatives of Air Electrodes in Zn–Air Batteries

**DOI:** 10.1002/advs.201700691

**Published:** 2018-01-22

**Authors:** Jing Pan, Yang Yang Xu, Huan Yang, Zehua Dong, Hongfang Liu, Bao Yu Xia

**Affiliations:** ^1^ Key Laboratory of Material Chemistry for Energy Conversion and Storage (Ministry of Education) Hubei Key Laboratory of Material Chemistry and Service Failure School of Chemistry and Chemical Engineering Wuhan National Laboratory for Optoelectronics Huazhong University of Science and Technology (HUST) 1037 Luoyu Road Wuhan 430074 P. R. China; ^2^ Shenzhen Institute of Huazhong University of Science and Technology Shenzhen 518000 P. R. China

**Keywords:** air electrode, architecture, electrocatalyst, flexible device, Zn–air battery

## Abstract

Zn–air batteries are becoming the promising power sources for portable and wearable electronic devices and hybrid/electric vehicles because of their high specific energy density and the low cost for next‐generation green and sustainable energy technologies. An air electrode integrated with an oxygen electrocatalyst is the most important component and inevitably determines the performance and cost of a Zn–air battery. This article presents exciting advances and challenges related to air electrodes and their relatives. After a brief introduction of the Zn–air battery, the architectures and oxygen electrocatalysts of air electrodes and relevant electrolytes are highlighted in primary and rechargeable types with different configurations, respectively. Moreover, the individual components and major issues of flexible Zn–air batteries are also highlighted, along with the strategies to enhance the battery performance. Finally, a perspective for design, preparation, and assembly of air electrodes is proposed for the future innovations of Zn–air batteries with high performance.

## 1 Introduction

Recently, sustainable development became a hot topic and major concern of the modern society, especially due to the great pressure of serious environmental issues and increasing energy demands.^1^ Renewable energy including solar, wind, waves, and hydropower are being the promising alternatives to replace the traditional fossil fuels to achieve the goal of green, economical, and sustainable society.^2^ However, their power output varies significantly over seasons, climates, and locations, and often mismatches the energy demands, even poisons gridding power.^3^ The effective and feasible solution to address the mismatch is to exploit energy storage and conversion technologies, and they are becoming the top priorities of current research for the scientific community, commercial companies, and national governments.^4^ As one of the most promising electrochemical energy technologies, lithium (Li)‐ion batteries lead the market of energy storage, especially consumer batteries in electronics and power batteries in hybrid/electric vehicles and stationary power plants.^5^ However, the insufficient energy densities of rechargeable Li‐ion batteries (200–250 Wh kg^−1^) limit their further development and applications.^6^

Metal–air batteries, whose theoretical energy density is even a few times more than the best performing Li‐ion batteries, are therefore being considered as an attractive solution and received more and more interests recently.^7^ Metal–air battery generates electricity via the redox reaction between metal at anode and oxygen in the air at a porous cathode, similar to the principles of fuel cells.^8^ The open cell structure of cathode is the paramount feature of metal–air batteries, which allows the continuous supply of oxygen from air.^9^ This feature directly endows the preference, especially a much higher theoretical energy density.^10^ Besides, the open structure also endows metal–air batteries many superiorities such as compact, light‐weight, and cost‐effective as this cathode replace the heavy and expensive constituents employed in Li‐ion batteries.^11^

There are various metal–air batteries according to different metal species used at anode.^12^ Among them, Li–air (oxygen) and Zn–air batteries are the most promising ones.^13^, ^14^ By now, Li–air batteries are the most discussed but controversial because of their combination of extreme high theoretical specific energy density as 5200 Wh kg^−1^ (including oxygen) and hazard potential.^15^ The hazard potential comes from explosive reactivity of lithium with air or water and ignites the flammable organic electrolyte used in most cases.^16^ Another inevitable drawback is the high cost (≈60 USD lb^−1^) and limited lithium resource occurring only in special natural mineral deposits in Australia and Chile.^17^ All above shortcomings of Li–air batteries limit their practical commercialization on large‐scale due to theses safety and economic issues.

Since the innate safety arise from using aqueous, nonflammable electrolytes and abundant global assets of zinc ore (Zn is the 4th most abundant in the earth crust, which is about 300 times greater than that of lithium),^18^ Zn–air batteries hold powerful potentials as the alternative to Li–air batteries, although the theoretical specific energy density of 1084 Wh kg^−1^ (including oxygen) is less than that of Li–air batteries, but still four times higher than those of current Li‐ion batteries.^19^ Besides, other advantages such as low cost and cheap (≈0.9 USD lb^−1^), low equilibrium potential, flat discharge voltage, long service life and environmental‐friendly further strongly guarantee the flourishing development of Zn–air batteries for the huge market of energy demands.^20^ In Zn–air battery, the most significant and complicated part is the air electrode integrated with gas diffusion layer and oxygen electrocatalyst layer.^21^ It is also closely related to the performance and cost which are the most outstanding technical challenges addressed for the target markets.^22^ However, the architectures of air electrode and wettability of components are often overlooked, which directly leads to a terrible performance of the whole cell even in case of employing highly active oxygen electrocatalysts.

Several previous excellent papers have reviewed the progress in the materials and systems of metal–air batteries,^23^ especially Li–air batteries,^24^ but few in Zn–air batteries.^25^ Nevertheless, in light of the increased research interest on Zn–air batteries, reviewing of current advances and future challenges is becoming highly necessary and significant. In this review, we summarize the continuous developments of Zn–air batteries, especially the exciting evolution of air electrodes over the past decades. After a brief introduction of Zn–air battery, the operation principles and relevant electrolytes are demonstrated. Then, a detailed discussion on the air electrode related issues for both primary and rechargeable Zn–air batteries are presented respectively. After that, regarding the fast development and demands of flexible devices, the technical issues of flexible Zn–air batteries with respect to air electrode, electrolyte, and assembly technique are also sequentially discussed. Finally, we will propose the understanding and perspective of the current trends and future challenges of air electrodes to address the intensive demands of Zn–air batteries. We hope this review would offer valuable insights for scientists and engineers to promote continuous innovations and commercialization of Zn–air batteries with high performance.

## 2 Zn–Air Batteries

Zn–air battery was initially proposed in 1878, while the silver wire acted as the air electrode.^26^ Few years later, a real gas diffusion electrode consisting porous carbon black and nickel current collector was reported in a so‐called Walker–Wilkins battery. Since the 1930s, a primary Zn–air battery was commercialized and was further applied in the hearing aids in 1970s.^27^ Now, its application has spread to seismic telemetry, railroad signaling, navigational buoys, remote communications, even electric vehicles and power grid.^28^, ^29^ However, hybrid/electric vehicles and power backup often need rechargeable batteries rather than primary ones.^30^

The evolution of rechargeable Zn–air battery is still hindered by nonuniform deposition of Zn and particularly the slow rates of oxygen evolution reaction (OER) and oxygen reduction reaction (ORR) at air electrode.^31^ There are many kinds of research focusing on Zn–air batteries between around 1975 and 2000, but the slow progress and the appearance of Li‐ion batteries sapped researchers' enthusiasm at the end of the 20th century.^32^ While in the recent few years, many improvements and huge energy demands restrike great interests in rechargeable Zn–air batteries.^33^ A lot of companies, such as EOS Energy Storage, Fluidic Energy, and ZincNyx Energy Solutions also joined the investigation and did a lot of excellent works.^32^ Nonetheless, as one of the promising alternatives for energy conversion and storage technologies, rechargeable Zn–air battery is still in the early stage. Therefore, extensive researches focusing on Zn–air batteries with outstanding electrochemical performance are growing in the flexible and wearable electronic devices for consumer batteries and rechargeable cells for power sources.

### 2.1 Configuration of a Zn–Air Battery

**Figure 1:**
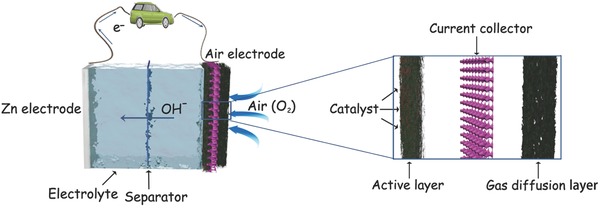
Schematic illustration of Zn–air battery and air electrode.

Zn–air battery consists of metal Zn electrode, membrane separator and air electrode, which are packaged together with the electrolyte, as illustrated in **Figure** **1**. The electricity is generated by the redox reaction between metal Zn anode and air cathode.^34^ Different parts of battery should satisfy different requirements.^35^ The zinc electrode which determines the capacity of the battery, should have a high activity and capacity for efficient recharging, and sustain the capacity over several hundred charge/discharge cycles.^36^ The separator should have a low electronic conductivity but a high ionic conductivity.^37^ The electrolyte should be appropriately active to Zn electrode, and have favorable conductivity as well as an excellent capability to sufficiently contact with air electrode.^38^ This review will focus on the air electrode, electrolyte, which is closely related to the air electrode, will also be briefly illustrated. The detailed description and challenges about Zn electrodes^28^, ^31^, ^39^ and separators^20^, ^40^ can refer to other excellent reviews.

### 2.2 Electrolyte of a Zn–Air Battery

Zn can react violently in acidic solution and cause severe anode corrosion, thus in the most cases alkaline rather than acid electrolyte is often employed.^41^ The most widely used aqueous alkaline electrolytes for Zn–air batteries are potassium hydroxide (KOH) and sodium hydroxide (NaOH). While, KOH is usually preferred over NaOH, due to its higher solubility of zinc salts, higher oxygen diffusion coefficients, and lower viscosity.^42^ The ionic conductivity of K^+^ (73.50 Ω^−1^ cm^2^ equiv^−1^) is also better than Na^+^ (50.11 Ω^−1^ cm^2^ equiv^−1^).^43^ It was further demonstrated on the Pt electrode directly, ORR process prefers KOH rather than NaOH solution in both thermodynamic and kinetic consideration.^44^ The concentration of KOH solution should also be kept in contemplation. A higher alkaline concentration to some extent leads to a higher ionic conductivity^45^ but it results in an increased viscosity thus decreases the transfer rate of hydroxyl ions, while 30 wt% KOH solution has a maximum ionic conductivity at room temperature.^46^ In addition, the concentration of KOH electrolyte can directly influence the ORR activity of catalysts, which is related to oxygen solubility and oxygen diffusion coefficients.^44^ The concentration of oxygen (*S*) and KOH (*C*) satisfy the relation of log*S* = −2.9–0.1746*C*, while the diffusivity of oxygen drops sharply with the increase of KOH concentration.^47^ Employing Pt electrocatalyst, it is demonstrated that ORR performance indeed frustrates as the KOH concentration increases. Thus, 0.1 m KOH solution is widely used for ORR tests of electrocatalysts during the RDE measurements. However, for the assembly of Zn–air battery, a much higher concentration of KOH electrolyte (6 m) is often utilized to ensure a high ionic conductivity and suppress hydrogen gas generated at the surface of Zn metals.^48^ As the low solubility and diffusivity of oxygen in KOH electrolyte with high concentration, gaseous oxygen can be utilized much more efficiently than the dissolved oxygen during the full cell operation and three‐phase ORR is of greater significance in ambient air than in pure oxygen.^48^ It desires to be mentioned that, recently, some soluble zinc salts are also added to KOH electrolyte to increase the rechargeable performance of Zn–air batteries, such as zinc acetate^49^, ^50^ and zinc chloride.^51^, ^52^

Aprotic electrolytes, especially ionic liquids (ILs),^53^ may also be a promising alternative to the aqueous electrolyte according to their nonflammable nature, low volatility, high chemical, electrochemical stability, good thermal stability, and intrinsic ionic conductivity.^54^ For Zn–air batteries, their negligible volatility nullifies the “drying‐out” of electrolyte.^55^ Moreover, ILs can also support the reversible deposition and dissolution of Zn and would mitigate the formation of zinc dendrites thus can be used in the secondary zinc–air batteries.^56^ The replacement of aqueous electrolyte by some proper ILs can also affect the performance of electrocatalysts through a different interface of electrode–electrolyte.^57^ It is believed that nonreactive oxygenated species would be prevented from absorbing on the surface of electrocatalysts and maintain the fluent mass transfer of reactants at the same time.^58^ For ORR process, a proton source is required, and it can be abstracted from the cations of ionic liquids in some instances.^59^ Thus, for practice, proton given additives are often added. The onset potential of ORR for electrocatalyst can be significantly influenced by the proton activity of ILs with or without additives.^60^ Utilizing ILs with optimal proton additives, ORR process can also be facilitated from two‐electron reduction pathway to four‐electron pathway on Pt.^61^ However, aprotic electrolytes are currently in their initial stages and much work need to be done for Zn–air batteries. For example, most of the ILs can wet polytetrafluoroethylene (PTFE) and fill the electrode pores, although they are usually too viscous to entirely soak air electrode.^39^ Nevertheless, aprotic electrolytes still have worse electrocatalytic activities than potassium hydroxide electrolyte because of their higher ionic resistance and different electrocatalytic mechanism.^62^

## 3 Primary Zn–Air Batteries

### 3.1 Reaction Process of a Primary Zn–Air Battery

The reaction process of a primary Zn–air battery includes Zn oxidation reaction happened at anode and ORR at cathode.^63^ As a whole reaction for full cell, oxygen spreads into air electrode and then is reduced to hydroxyl ions at active catalyst layer, after that, the resultant hydroxyl ions migrate to anode and combine with zinc ions produced to form soluble zincate ions (Zn(OH)_4_^2−^).^64^ When Zn(OH)_4_^2−^ reaches its saturation concentration, it will be decomposed to ZnO species.^39^, ^65^ The chemical reaction equations are listed below. Anode: Zn → Zn^2+^ + 2e^−^; Zn^2+^ + 4OH^−^ → Zn(OH)_4_^2−^; Zn(OH)_4_^2−^ → ZnO + H_2_O + 2OH^−^. Total: Zn + 2OH^−^ → ZnO + H_2_O + 2e^−^ (*E*_0_ = −1.25 V). Cathode: O_2_ + 2H_2_O + 4e^−^ → 4OH^−^ (*E*_0_ = 0.4 V). Overall reaction is 2Zn + O_2_ → 2ZnO (*E*_0_ = 1.65 V). Here *E*_0_ represents the standard electrode potential with respect to the standard hydrogen electrode.^66^ However, the practical working voltages are usually lower than 1.2 V, much less than standard potential (1.65 V) in consideration of the internal loss of battery, resulted from the activation, ohmic polarization and concentration loss.^67^

### 3.2 Air Electrode Architecture of a Primary Zn–Air Battery

Conventional air electrode is composed of three major components: current collector, gas diffusion layer, and active catalyst layer (Figure 1). The current collector is usually a conductive metal mesh, such as nickel foam^68^ and stainless steel.^69^ The gas diffusion layer, which is a channel for oxygen, should have a highly effective surface area which is preferable for the gas transfer and must be hydrophobic for the air contact while avoiding the leakage of electrolyte.^70^ The mixture of porous carbon materials and PTFE is often employed as the gas diffusion layer.^71^ The active layer is where ORR takes place and is crucial for the effective working of Zn–air batteries.^72^ In most cases, the active layer covers on the surface of current collector and contacts with electrolyte, while the gas diffusion layer lies on the reverse side and faces to the open air, and the current collector is placed in the middle of active layer and gas diffusion layer and then forms a sandwich structure.^73^ As oxygen has low solubility and diffusivity in most of the electrolytes, oxygen in the ORR process is mainly in the form of gas phase, thus boundary with a high‐surface area between triple active phases: gas (air), liquid (electrolyte), and solid (catalyst) is very crucial for an air electrode.^74^ That is why a dimensional porous architecture is favorable for air electrode.^75^

Except for gas diffusion layer, the active layer should also contain a porous substrate to supply enough space and react with oxygen at the surface of catalysts.^76^ Supporting materials are often employed to increase the utilization, enhance the activity and prolong the survival life of catalysts. Thus, high specific surface area, porous structure and abundant active side chain are needed to promote the good interaction of gaseous oxygen in electrolyte solution on the surface of catalysts.^77^ Moreover, the excellent conductivity, good stability, and corrosion/oxidation resistance are also important for supporting materials as the electron transfer happens during the long‐term harsh electrochemical process.^25^ Consequently, porous nanocarbons have also been demonstrated to be the most widely used supporting materials because of their distinctive physical and chemical advantages together abundant nature source and low‐cost manufacture.^78^ For example, graphene‐based composites have been applied in the catalyst layers.^79^ Furthermore, in order to make the catalysts tightly stick to carbon substrates, some polymer binders are also often introduced.^80^, ^81^

Since the crucial ORR mainly occurs at the triple phase zone (gaseous oxygen/solid electrocatalyst/liquid electrolyte),^82^ to meet the rigorous requirements of ORR, the excellent contact of oxygen and electrolyte on the surface of electrocatalyst is necessary and significant when constructing this triple phase boundary. While the wettability (hydrophobicity/hydrophilicity) of the components of air electrode with the electrolyte should be in charge of the sufficient contact.^13^ In addition, Zn–air batteries are very sensitive to the humidity of surrounding environment, the well‐balanced hydrophobicity/hydrophilicity can alleviate the evaporation loss and resist flooding of electrolyte.^83^ To realize the optimized wettability, the side contacting with electrolyte (active catalyst layer) should be hydrophilic, while the other side facing to air (gas diffusion layer) should be hydrophobic.^84^ The wettability of air electrode is often realized by the use of hydrophobic organic polymer particles such as PTFE,^85^ which is capable of water repellent properties and high chemical stability.^86^ PTFE is a synthetic fluoropolymer of tetrafluoroethylene and a kind of Teflon which is a registered trademark of DuPont company. The appearing of Teflon makes Zn–air batteries commercially feasible and allows oxygen electrode to operate efficiently.^87^ Actually, Teflon also contains other fluorinated ethylene propylene (FEP),^88^ but they are not so widely used in Zn–air batteries as PTFE.

An integrated air electrode for Zn–air battery is very complicated, hence, simple configurations are widely employed in the practical application.^89^ In a large proportion of cases, the diffusion layer is absent as porous carbon substrate in the active layer has a large specific surface area, which would effectively diffuse oxygen from the outer atmosphere into the cell.^90^ One typical method is to mix catalyst, porous carbon materials, polymer binder (PTFE) and press slurry onto a chosen current collector (nickel foam or other metal grids).^91^ In this design, PTFE is not only the hydrophobic coating but also a binder. The wettability of air electrode is determined by the proportion of carbon supports and PTFE used at different fabrication conditions. The proper PTFE content is 30–70 wt%,^92^ so that the active layer could be only partly wetted by the electrolyte.^93^ However, the different wettability of air electrode is of great importance, the absence of gas diffusion layer would inevitably affect the battery performance.

### 3.3 Oxygen Electrocatalysts of a Primary Zn–Air Battery

The operation of primary Zn–air batteries is deadly depends on ORR process at air cathode, thus the key component of air electrode is ORR electrocatalyst.^94^ However, the sluggish kinetics of ORR results in high overpotentials which would lower energy efficiency, and finally limit the output performance of primary batteries.^95^ Therefore, highly efficient ORR electrocatalysts are obligatory in the good operation of Zn–air batteries.^96^ The requirements of desired properties for efficient electrocatalysts include high active site density and uniform distribution for high ORR onset potential and high catalytic activity,^97^, ^98^ high surface area and sufficient porous structure for the sufficient mass transfer pathways^99^ and enhanced electrode kinetics,^100^ robust architecture for the chemical and mechanical stability for high durability, high mass and volumetric activities, and finally the abundant resource with low‐cost.^101^ However, most electrocatalysts are far from acceptability, thus the practical energy densities of existing Zn–air cells typically are far less and only 40–50% of the theoretical density.^102^

Basically, there are two standard ORR pathways takes place in alkaline solutions, direct four‐electron pathway (H_2_O) and indirect two‐electron pathway (H_2_O_2_).^103^ In the former, an oxygen molecule receives 4 electrons and is reduced to 4 OH^−^ (O_2_ + 2H_2_O + 4e^−^ → 4OH^−^, 0.4 V). In the latter, oxygen molecules are first reduced to intermediate H_2_O_2_, and through a further reduction to form OH^−^ (O_2_ + H_2_O + 2e^−^ → HO_2_^−^ + OH^−^ (−0.07 V), HO_2_^−^ + H_2_O + 2e^−^ → 3OH^−^ (0.87 V)).^104^ Obviously, the 4‐electron mechanism is more efficient and favorable for Zn–air batteries, which would avoid the premature degradation of electrochemical cell caused by corrosion/oxidation of carbon supports and other materials by peroxide.^105^ Plentiful materials have appeared in the area of ORR electrocatalysts, including noble metals and their alloys, transitional metals, metal oxides/chalcogenides/carbides/nitrides, carbon nanomaterials, and their composites.^106^ However, different reaction mechanisms may happen on the surface of different catalysts.^107^ The 2‐electron reduction path is favored on most of nanocarbons^108^, ^109^, ^110^ and transitional metal based composites,^48^, ^111^, ^112^ while the direct 4‐electron reduction is favored on noble metal‐based catalysts.^113^, ^114^ Various ORR pathways exist on the transition‐metal based electrocatalysts, which depend on the molecular composition and specific crystal structures, even the experimental conditions.

Benefit from the similar principles, most of the oxygen catalysts employed in alkaline fuel cells and other alkaline metal–air batteries could also be served in Zn–air batteries.^115^ Noble metals and alloys,^116^ especially platinum (Pt),^117^ have been recognized as the most active electrocatalysts for ORR.^118^, ^119^ For example, PtCu nanocage was employed as an efficient ORR electrocatalyst for primary Zn–air battery.^120^ Alloying Pt with other appropriate metals even coupled with supporting materials can effectively improve the utilization and reduction of Pt usages,^121^ but the limited reserves, high price and poor stability of noble metals still hinders their widespread implementation in Zn–air batteries.^122^ Consequently, tremendous efforts are devoted to exploring, designing, and preparing nonprecious metal alternatives with high performance.^123^ For instance, MnO_2_ is frequently used as ORR catalysts.^124^, ^125^, ^126^ In Duracell hearing‐aid batteries, γ‐MnO_2_ was selected and this battery exhibited a very high energy densities of 400 Wh kg^−1^.^39^

**Figure 2:**
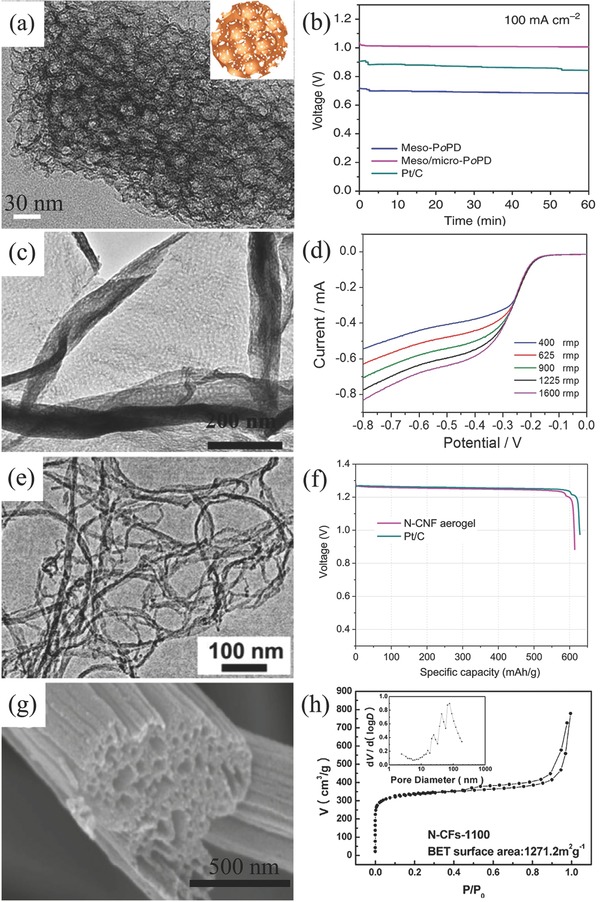
TEM image (a) and galvanostatic discharge curves of hierarchical N‐doped porous carbon (b). Reproduced with permission.^130^ Copyright 2014, Nature. TEM image (c) and RDE voltammograms (d) of nanoporous N‐doped carbon/graphene. Reproduced with permission.^131^ Copyright 2012, RSC. TEM image (e) and galvanostatic discharge curves (f) of Zn–air batteries with porous N‐doped carbon fiber. Reproduced with permission.^132^ Copyright 2015, Elsevier. SEM image g) and N_2_ sorption isotherm and pore size distribution h) of porous N‐doped fiber. Reproduced with permission.^133^ Copyright 2013, Elsevier.

As mentioned above, carbon‐based materials are satisfactory supports. After heteroatoms doping (N, S, P, even transitional metal atoms), they also have potentials as the ORR electrocatalysts.^127^ The discovery of N‐doped carbon nanotube (CNT) as ORR catalyst inspires a boom of researches toward nanocarbon based electrocatalysts,^128^ their application in Zn–air batteries also spring up and much progress have been made.^129^ For example, Liang and his colleague reported a kind of hierarchical porous N‐doped carbon material with ultrahigh ORR catalytic activity even better than that of Pt/C (**Figure** **2**a).^130^ When assembled to Zn–air battery, this material behaved better than Pt/C especially at a high current density of 100 mA cm^−2^ (Figure 2b).^130^ Besides, N‐doped porous nanocarbons/graphene composite also exhibited a similar 4e ORR pathways as Pt/C (Figure 2c,d).^131^ Another battery sample assembled by N‐doped carbon nanofiber aerogel in Figure 2e has a specific capacity of ≈615 mA h g_Zn_^−1^ at a discharged current density of 10 mA cm^−2^ (Figure 2f).^132^ The characteristic feature of carbon‐based catalysts is a high specific surface area, for instance, 1271 m^2^ g^−1^ for N‐doped carbon fiber (Figure 2g,h).^133^ Moreover, the doped porous carbon materials derived from metal–organic frameworks (MOFs) recently attracted progressive attention, benefiting from the abundant composition and excellent porous structure.^134^ For example, Chen and co‐workers employed Cu‐doped ZIF‐8 to synthesize Cu nanoparticles embedded in N‐doped mesoporous carbon polyhedron, the maximal power density of Zn–air battery assembled by this material was high to 132 mW cm^−2^.^135^

Some current ORR electrocatalysts employed in Zn–air batteries have been summarized in **Table** **1**. It can be seen that noble metal‐based catalysts still exhibit a relatively better performance. However, the catalytic activity of non‐noble metal oxide catalysts can be enhanced by proper morphology, doping or cooperating with each other. Most nanocarbon‐based catalysts have the number of the electron transfer per oxygen molecule (*n*) lower than 4, but the lightweight and good conductivity can result in a relatively higher energy density. In this consideration, many researchers are focusing on cooperating different kinds of catalysts together to achieve the enhanced performance while lowering the cost of catalysts.

**Table 1:** Summary of oxygen catalysts performed in primary Zn–air batteries

Catalystsa)	ORR activity	Battery performancea)	Ref.
Cu‐Pt nanocage	Onset potential: 0.95 V; Tafel slope: 69.94 mV dec^−1^; *n* ≈ 4	Specific capacity: 560 mA h g_Zn_^−1^; energy density: 728 Wh kg_Zn_^−1^	120
Electrolytic MnO_2_	–	Power density: 141.8 mW cm^−2^	107
Ag_4_Bi_2_O_5_/MnO_2_	*n* ≈ 3.7–3.8	Discharge time: 225 h at 120 mA cm^−2^; power density: 250 mW cm^−2^	126
2D porous carbon	Onset potential: 0.930 V	Open‐circuit voltage: 1.48–1.52 V; specific capacity: 750 mA h g^−1^	108
Graphene composite	*n* = 3.2–3.6	Power density: 70 mW cm^−2^	131
N‐microporous carbon	*n* = 3.0–3.7	Discharge time: 3500 s at 50 mA cm^−2^	110
N‐doped carbon fiber	*n* = 3.7–3.8	Power densities: 194 mW cm^−2^	133
N‐doped carbon nanofiber aerogel	Half‐wave: 0.80 V vs RHE; *n* = 3.97	Specific capacity: ≈615 mA h g^−1^; gravimetric energy density: ≈760 Wh kg^−1^ at 10 mA cm^−2^	132
N‐CNTs	*n* = 4	Power density: 70 mW cm^−2^	76
S/N‐carbon nanosheets	*n* = 3.96	Power density: 252 mW cm^−2^	109
Ag/C	*n* ≈ 3	Power density: 34 mW cm^−2^	116
Ag/CNCT	–	Specific energy density: 300 W h kg^−1^	160
Pyrolyzed CoTMPP	*n* = 2.92	Reach above 120 mA cm^−2^ at 1 V	98
Pyrolyzed FeCo‐EDA (FeCo‐N‐C)	Onset potential: −0.05 V vs SCE	Power density: 232 mW cm^−2^	97
Graphitic carbon@CuFe	Tafel slope: 90 mV dec^−1^	Power density: 212 mW cm^−2^	99
Amide‐CoO*_x_*/C composite	Onset potential: ≈0.86 V vs RHE	Power density: 100–123 mW cm^−2^	112
RGO–IL/Mn_3_O_4_	*n* = 2.75	Power density: 120 mW cm^−2^	112
MnO*_x_*/Ketjblack	Onset potential: −0.05 V vs Hg/HgO	Power density: 190 mW cm^−2^	111
Ni@MnO*_x_*/C	Onset potential: −0.177 V vs Hg/HgO; *n* = 3.83	Power density: 122 mW cm^−2^	124
α‐MnO_2_/XC‐72	*n* = 3.8	Power density: 61.5 mW cm^−2^	125
Mn*_x_*Co_3−_ *_x_*O_4_/N‐Ketjen black carbon	*n* ≈ 4.1; Tafel slope ≈ 56 mV dec^−1^	–	48
C‐PDA/Fe_3_O_4_	Onset potential: −0.14 V	Stable discharge voltage for over 200 h	100

^a)^ The detail test condition refers to the primary references.

## 4 Rechargeable Zn–Air Batteries

### 4.1 Reactions in a Rechargeable Zn–Air Battery

For rechargeable Zn–air battery, discharge process is just like the primary ones, while charge process is reversible with discharge process.^136^ During the charge process, reactions happening at the cathode is the reduction of ZnO to metallic Zn, which is ZnO + H_2_O + 2OH^−^ → Zn(OH)_4_^2−^ and followed by Zn(OH)_4_^2−^ + 2e^−^ → Zn + 4OH^−^. Meanwhile, the oxidation of oxygen species (hydroxyl) to oxygen takes place at the anode, 2OH^−^ → ½ O_2_ + H_2_O + 2e^−^. Thus, the overall reaction of charging is 2ZnO → 2Zn + O_2_. For primary Zn–air battery, ORR is the only functional reaction during the discharge process and key rate‐limiting step happened in the air electrode. While, for rechargeable ones, OER is another functional reaction for charging process.^137^ Thus, discharging and charging processes are promoted by the electrocatalytic ORR and OER, respectively.^138^

### 4.2 Configurations of a Rechargeable Zn–Air Battery

There are two types of rechargeable Zn–air batteries, mechanical and electrical.^113^ The prominent difference between them is that external recharging in mechanical ones by removing and replacing the discharged anodes or products such as zinc oxides and zincates, while the discharge/charge process takes place within the electrically rechargeable battery configuration.^139^ As the high cost of building the network for zinc recharging and supplying stations, mechanically rechargeable batteries are not widely used. In this critical review, we put emphasis on the electrical rechargeable Zn–air batteries.

**Figure 3:**
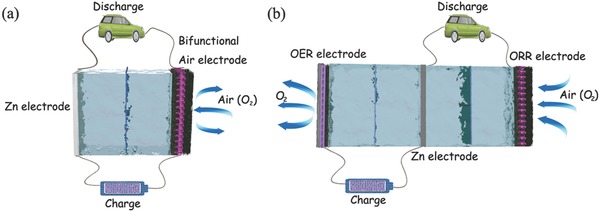
Schematic configurations of rechargeable Zn–air battery with two‐electrode (a) and trielectrode (b).

For an electrically rechargeable Zn–air battery, the basic and most widely used configuration is the two‐electrode model.^140^ It is similar to primary Zn–air battery, while only the unifunctional ORR air‐cathode is replaced by a bifunctional electrode integrated with a bifunctional oxygen electrocatalyst or mixture of ORR and OER catalysts (**Figure** **3**a). Thus, oxygen electrochemical processes (ORR/OER) happen at the bifunctional air electrode during the discharging/charging processes respectively.^141^ However, it is theoretically in a short cycle life because ORR catalysts would be deactivated during the charging process at high voltages. In the discharging process, open circuit voltage of Zn–air battery is usually around 1.2 V. However, charging voltage needs to be raised to ≈2.0 V even higher due to the large overpotential of OER.^39^ The high potentials would cause the oxidation and corrosion of oxygen electrocatalysts. Moreover, porous structure is too fragile to suffer the gas generated (OER) in the charging process, which would result in the mechanical breakdown and catalyst loss of the electrode, followed by the death of battery.^142^

**Figure 4:**
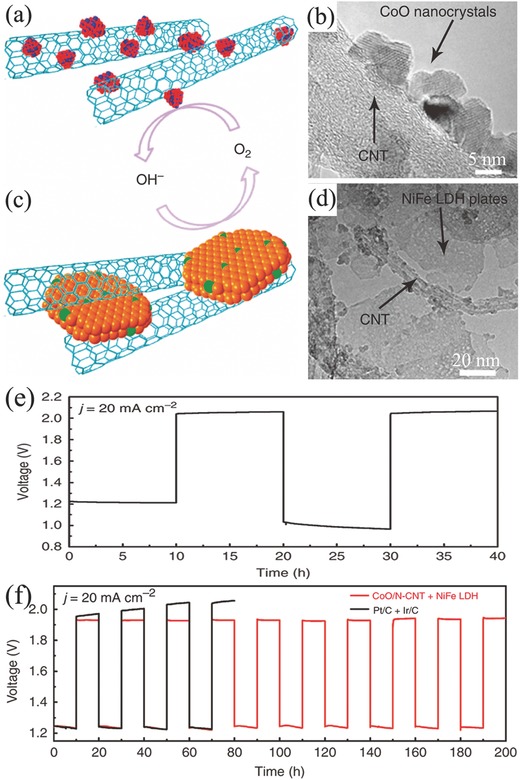
Schematic structure (a) and TEM image (b) of CoO/N‐CNT hybrid. Schematic structure (c) and TEM image (d) of NiFe LDH/CNT hybrid. Cycling performance for two‐electrode configuration (e) and trielectrode configuration (f) rechargeable Zn–air battery. Reproduced with permission.^142^ Copyright 2013, Nature.

From the advantages of primary Zn–air battery and two‐electrode rechargeable Zn‐battery, a trielectrode configuration is then developed to solve this problem. The trielectrode configuration consists of two separated air electrodes corresponding to ORR and OER respectively, while Zn electrode is placed between ORR electrode and OER electrode (Figure 3b). ORR electrode connects with Zn electrode during discharging, while OER electrode is connected with Zn electrode during charging.^143^ Dai and co‐workers developed rechargeable Zn–air batteries in both two‐electrode and trielectrode configurations.^142^ CoO/carbon nanotube (CNT) hybrid and NiFe layered double hydroxide (LDH)/CNT composite were employed as ORR and OER catalyst, respectively (**Figure** **4**a–d). The air electrode was prepared by loading a simple mixture of ORR and OER catalyst on a Teflon‐treated carbon fiber paper with Nafion and was also paired with a Zn electrode in a two‐electrode system. The assembled battery demonstrated only a stable cycling performance when charging/discharging at low current densities (5–10 mA cm^−2^). However, the discharging overpotential of 200–250 mV after the second cycle was much obvious than the initial due to the partial oxidation and inactivation of oxygen electrocatalyst in the first charging process. When ORR and OER electrocatalysts were respectively loaded onto the separated ORR and OER electrodes, integrating with a porous Ni foam as current collector, the electrochemical properties of Zn–air battery in a trielectrode configuration was improved remarkably. Especially, the battery durability was improved respect to the two‐electrode configuration as it demonstrated a high cycling stability when repeatedly charged or discharged for a total of 200 h (20 h per cycle) at 20 mA cm^−2^. The overpotential increased was only 20 mV after 100 h operation in the case of anodically biased at 20 mA cm^−2^ (Figure 4e,f).

Although the trielectrode configuration ensures a higher battery cycling stability than two‐electrode configuration, it unavoidably increases the volume and weight of batteries, thus reducing the volumetric energy as well as power density. Hence, the simple design of two‐electrode configuration is still more widely used. To overcome the drawbacks of two‐electrode configuration, a number of researches were devoted to preparing robust bifunctional oxygen electrocatalysts or bifunctional air electrodes. But until now, it is still a big challenge as most of the current bifunctional oxygen electrocatalysts exhibit uneven activity for ORR and OER, or limited stability, which will be reviewed in the following sections.^144^

### 4.3 Air Electrode for a Rechargeable Zn–Air Battery

Actually, air electrode determines the type and configuration of Zn–air battery, further the physical structure of air cathode seriously influence the final electrochemical performance of the battery. An air electrode optimally executes multiple functions including O_2_ diffusion, ion transport, electron transfer, electrocatalytic activity, and accommodate precipitate formation.^39^, ^145^ Thus, similar to primary ones, rechargeable Zn–air batteries also require high surface area to load and anchor oxygen electrocatalysts at catalytic active layer and appropriate pore channel for the efficient mass transfer and oxygen diffusion. As the role of oxygen electrocatalyst is to promote ORR and/or OER during the discharging–charging, and the electrochemical process happens just at the triple phase zone between liquid electrolyte and solid oxygen electrocatalysts/supports and gas oxygen reactant, the sufficient wettability of active catalyst layer allows the oxygen electrochemical reactions happen at the surface/interface of triple phase zone. Liu and co‐workers tuned the wettability of carbon nanotube arrays through adjusting the preparation process for efficient bifunctional electrocatalyst for Zn–air battery.^146^ Getting away the block of polymers, this approach demonstrated a better electrochemical performance. Moreover, the excellent interaction between electrocatalyst with substrate and the conductivity of whole electrode is also needed to meet the requirements for fast electron transfer and low interfacial resistance.

Compared to primary ones in practice, the significant change of air electrode in electrically rechargeable Zn–air batteries is that ORR electrocatalysts are transformed to bifunctional oxygen (OER/ORR) electrocatalysts, thus the same requirements as the primary batteries needed but not limited to these. OER happens during the charging of rechargeable Zn–air batteries, thus robust structure rather than fragile porous structure is more suitable for oxygen gas involved evolution reaction.^143^ Moreover, except the physical characters required, other properties like mechanical, thermal, electrochemical, and chemical stability are also very important to the stable operation of rechargeable batteries. The widely used carbon supports in ORR electrodes suffer from serious degradation under high potentials of OER process.^147^ It is reported that the potential only above 0.207 V can make carbon materials be thermodynamically corroded to carbon dioxide. This oxidation can become even obvious when a noticeable current density of oxygen evolution is often generated over 1.5 V, even RuO_2_ is employed.^148^ Thus, the anticorrosion/oxidization of carbon materials could be enhanced and utilized as low as possible in OER electrode.

Except the physical and chemical properties required in the electrode, the limited space in electronics and controlling on the weight is also significant to improve the quality and performance of batteries. These are obviously depending on the preparation techniques. The traditional fabrication via “brick‐and‐mortar” route requires the addition of many ancillary and inactive additives including polymeric binders and catalyst supports. These additives may not only contribute an excess weight to the final electrode (≈40%),^149^ but also compromises the electrochemical cell performance by the increased interfacial resistance caused by the insulating polymeric binders and the reduction of accessible active sites. The decomposition of additives during the reactions would also cause some catalysts fall off from the electrode surfaces.^150^ What's more, the additives such as PTFE may be oxidized and be failed over long operation times.^151^, ^152^ As a consequence, air electrodes with a minimized use of ancillary additives are serious in demand.

**Figure 5:**
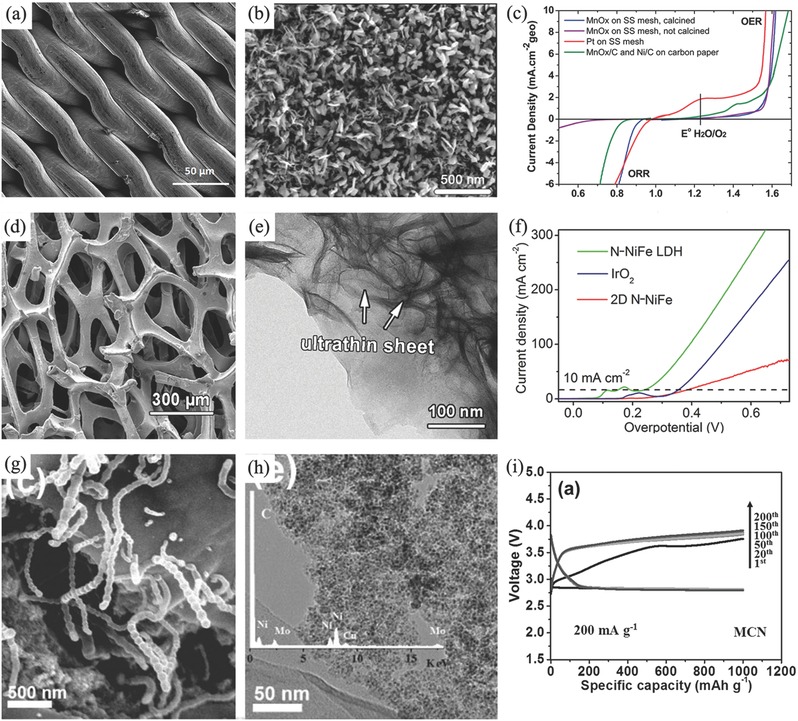
SEM images of bare (a) and MnO*_x_* deposited stainless steel mesh (b); linear sweep voltammogram (LSV) curves (c) of MnO*_x_* deposited stainless steel mesh. Reproduced with permission.^154^ Copyright 2014, RSC. SEM (d) and TEM (e) images, LSV plots (f) of OER catalytic properties of N‐NiFe LDH. Reproduced with permission.^155^ Copyright 2015, Wiley. SEM (g), TEM (h) images, and cycle performance (i) of MCN at current density of 200 mA g^−1^. Reproduced with permission.^156^ Copyright 2016, Wiley.

A characteristic binder‐free air electrode is made by the direct growth of carbon nanofiber arrays on an anodized aluminum oxide (AAO) substrate which is covered by thin layers of Ta and Fe. Fe layer served to catalyze the growth of carbon nanofiber arrays, while Ta layer is used as the conductive underlayer.^153^ However, AAO substrate is inactive. Consequently, the direct growth of oxygen electrocatalysts on the conductive current collector becomes the straightforward way to get an entirely binder‐free air electrode. In addition of binder‐free, this integration design can enhance the electron transfer between active layer and current collector. Furthermore, if metal materials are employed as the current collectors, the formed carbon‐free electrodes would also avoid the subsequent negative issues from carbon corrosion/oxidation at high potentials. For example, Jaramillo's group employed stainless steel (SS) as current collector and directly electrodeposited manganese oxide (MnO*_x_*) as oxygen electrocatalyst (**Figure** **5**a–c). The integrated MnO*_x_*/SS air electrode displayed an excellent oxygen electrocatalytic activity and stability toward ORR and OER, which was almost equal to MnO*_x_* and Ni catalysts deposited onto a carbon paper substrate.^154^ Qiao and co‐workers developed an in situ method to fabricate a multifunctional electrode consisting of N‐doped NiFe double layer hydroxides (LDH) on 3D Ni foam (Figure 5d–f). This integrated electrode demonstrated an outstanding OER catalytic activity and onset overpotential was only 0.21 V and thus was proposed as a good candidate for rechargeable Zn–air battery.^155^ Another example is a hybrid electrode of Ni foam supported carbon‐wrapped Mo_2_C nanoparticles/carbon nanotubes (Figure 5g–i). In this composite, porous carbon skeleton together with carbon nanotubes protruded from the composite forms a special 3D structure and gives a good access to oxygen gas evolution.^156^

**Figure 6:**
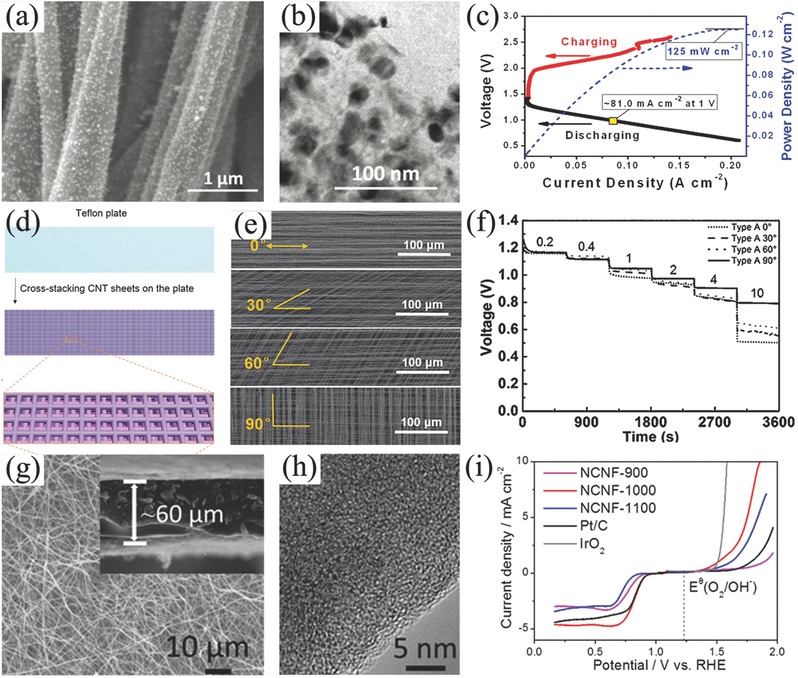
SEM (a), TEM (b) images, and battery performance (c) of C‐CoPAN900 mat based Zn–air battery. Reproduced with permission.^163^ Copyright 2015, RSC. Schematic preparation of CNT sheet based air electrode (d), SEM images of CNT sheets with different cross‐stacking angles of 0°, 30°, 60°, and 90° (e), rate discharge curves of flexible Zn–air batteries with different cross‐stacking angles of 0°, 30°, 60°, and 90° at different current densities (f). Reproduced with permission.^164^ Copyright 2015, Wiley. SEM (g), TEM (h) image, and oxygen catalytic properties of NCNF‐1000 (5 mV s^−1^) (i). Reproduced with permission.^167^ Copyright 2016, Wiley.

Actually, metallic mesh current collector still contributes a large proportion of the weight of traditional air electrode. Graphitic carbons such as carbon paper and carbon cloth as current collectors can dramatically reduce the weight of electrodes, thus they are widely applied to other energy devices such as Li‐ion batteries,^157^ supercapacitors,^158^ and Li–S batteries.^159^ Combination with nanotechnology, engineering graphitic carbon materials with active materials in free‐standing and porous structure is promising to maximize the utilization of current collectors.^160^, ^161^, ^162^ For example, a binder‐free air electrode made by Co_3_O_4_ nanoparticles decorated on carbon nanofiber mat was developed by thermally treating electrospun Co(II)‐containing polyacrylonitrile fibers, without any further modifications (**Figure** **6**a,b).^163^ The integrated Zn–air battery exhibited a small discharge/charge potential gap of 0.7 V at 2 mA cm^−2^, and the power density achieved 125 mW cm^−2^ (Figure 6c), which was ≈4 times higher than that of Zn–air battery equipped by the corresponding conventional air electrode (29 mW cm^−2^). Moreover, this battery also exhibited a better stability and cycling performance compared to battery assembled with Pt/C based air‐cathode. With the introduction of N‐doped graphitic carbon materials as ORR and/or OER electrocatalysts, the structure of air electrode can be further simplified. Peng and co‐workers developed a kind of carbon nanotube sheets as air electrode for flexible and stretchable fiber‐shaped Zn–air battery, in which CNT/RuO_2_ composites simultaneously worked as gas diffusion layer, catalyst layer, and current collector at the same time (Figure 6d,e).^164^ This assembled battery exhibited an enhanced discharge voltage plateaus especially at high current densities. Employing this simple air electrode, flexible fiber‐shaped Zn–air battery can be discharged/charged at 1.0 V at a high current density of 1 A g^−1^ (Figure 6f). After this, this design approach was further employed to assemble flexible Al–air batteries in a fiber‐shaped.^165^ Besides, the free‐standing graphene‐based composites could be used as air electrode if integrated with conductive Ag nanowires. For example, Zn–air battery employed this 3D graphene/Ag nanowires cathode can permit an ultrahigh discharge rate of up to 300 mA cm^−2^.^166^

Obviously, doped‐ or modified‐graphitic carbon materials with porous structure can be as diffusion layers and electrocatalyst supports, high electrochemical performance can be as bifunctional oxygen electrocatalyst and good electrical conductivity can be as current collector, thus they have great potentials as the alternative air electrode simultaneously and further simplify from three components to only one part even one material if it can also be 3D and self‐standing. Liu and co‐workers prepared nanoporous N‐doped carbon nanofiber films after a thermal treatment of electrospun polyimide film (Figure 6g,h). This free‐standing film demonstrated many advantages including high specific surface area of 1249 m^2^ g^−1^, electrical conductivity of 147 S m^−1^, moderate tensile strength of 1.89 MPa and tensile modulus of 0.31 GPa. Importantly, this film also had a bifunctional catalytic activity for ORR (onset potential of 0.97 V and current density of 4.7 mA cm^−2^) and OER (onset potential of 1.43 V and potential of 1.84 V to achieve 10 mA cm^−2^, Figure 6i).^167^ Based on this extraordinary film, air electrode can be simply applied on primary liquid Zn–air battery, rechargeable liquid Zn–air battery and flexible all‐solid‐state rechargeable Zn–air battery, respectively. However, this kind of simple air electrode is very scarce. Although vertically aligned carbon nanotubes (VACNT)/graphene paper,^168^ 3D VACNT‐graphene architectures,^169^ all carbon nanotube ultrathin films,^170^ and free‐standing CNT/graphene film are all realized,^171^ few of them are investigated in the area of rechargeable Zn–air batteries. In addition, nanocarbons such as graphene and CNT are proved either hydrophobic or hydrophilic.^172^ And the wettability of nanocarbons in the simplified air electrodes has been seldom discussed in practical operation. Thus, it would be proposed as a new promising method to promote the properties of this simple air electrode, and there is still enormous space for improvements in the electrochemical properties of Zn–air batteries in the aspects of integrated air electrode.

### 4.4 Oxygen Electrocatalyst for a Rechargeable Zn–Air Battery

Highly active and robust oxygen electrocatalysts are crucial for power, energy densities and energy efficiencies of Zn–air batteries,^173^, ^174^ and they are mostly focused on to develop high‐performance rechargeable Zn–air batteries.^175^, ^176^ However, ORR and OER process are so different that it is extremely difficult for one catalyst to satisfy the request for both oxygen electrochemical reactions.^177^ For Pt catalyst example, the equimolar ratio of primary oxide (Pt—OH) and surface oxide (Pt=O) is critical to ORR, but the formation of irreversible Pt=O decrease its catalytic activity toward OER.^178^ Oppositely, IrO_2_, RuO_2_ are effective for OER but not as active for ORR.^179^ Although nanotechnologies and nanomaterials remarkably increase the catalytic activity and reduce the usage of precious metals involved,^180^, ^181^ they are still limited due to the scarce assets and high cost,^182^, ^183^, ^184^ thus huge economic motivation and extensive scientific interests stimulate the exploration of cheap and earth‐abundant non‐noble metal alternatives to promote further development and commercialization of Zn–air batteries.^119^, ^185^, ^186^

**Figure 7:**
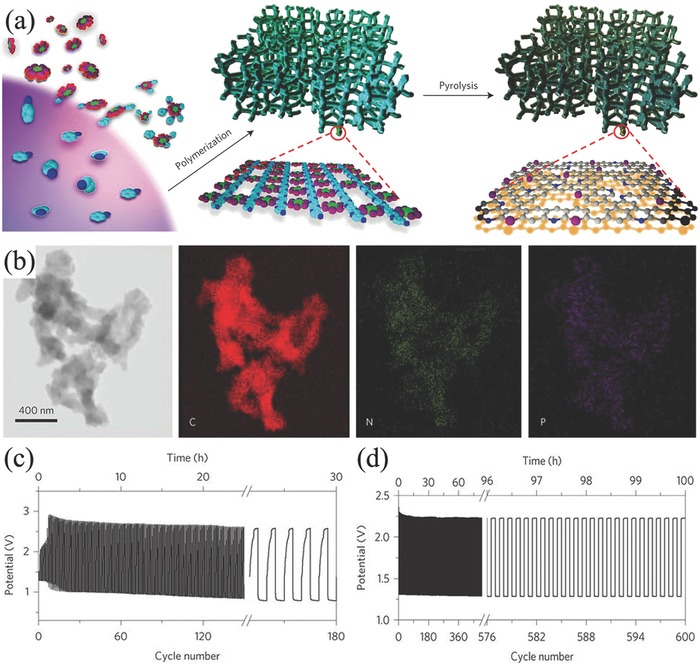
Schematic illustration of the synthesis method (a) and TEM images with corresponding element mapping (b) of N, P co‐doped mesoporous carbon foams. Cycling performance of rechargeable Zn–air batteries corresponding two‐electrode configuration (c), and trielectrode configuration (d) at current density of 2 mA cm^−2^. Reproduced with permission.^188^ Copyright 2015, Nature.

In consideration to the advantages of tri‐electrode configuration in both separated ORR and OER electrodes, employing the same carbon electrocatalysts in the tri‐electrode configuration leads to a better cycle performance than the corresponding two‐electrode configurations.^187^ For example, primary battery assembled by N,P co‐doped carbon foam demonstrated an open‐circuit potential of 1.48 V and energy density of 835 Wh kg_Zn_^−1^ (**Figure** **7**a,b).^188^ When assembled to rechargeable batteries in the form of tri‐electrode configuration, the cell demonstrated an excellent stability of 600 charge/discharge cycles for 100 h (Figure 7d), which was almost equal to that of Pt/C and RuO_2_ in tri‐electrode and better than same carbon electrocatalyst in the two‐electrode configuration of 180 cycles at 2 mA cm^−2^ (Figure 7c).

**Figure 8:**
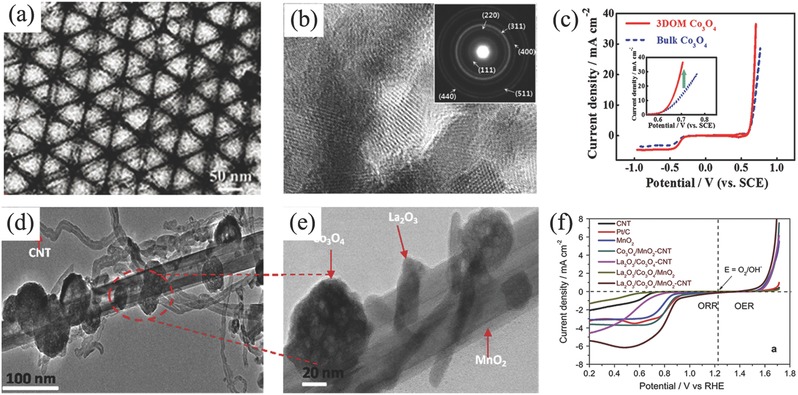
a,b) TEM images and c) oxygen electrochemical performance of 3D ordered mesoporous Co_3_O_4_. Reproduced with permission.^195^ Copyright 2016, Wiley. d,e) TEM images and f) corresponding ORR and OER polarization curves of La_2_O_3_/Co_3_O_4_/MnO_2_‐CNTs hybrid catalyst. Reproduced with permission.^198^ Copyright 2016, Elsevier.

In spite of the capacity of the mixture of ORR and OER electrocatalyst employed in the air electrode, tremendous efforts have been devoted to developing bifunctional oxygen electrocatalysts.^189^ The most concerns about nonprecious oxygen electrocatalysts are transitional metal oxides such as spinel cobalt oxides (Co_3_O_4_).^190^, ^191^ However, most of them are of good OER catalytic activity but weak ORR catalytic activity.^192^, ^193^ For example, Manthiram and co‐workers employed Ni foam supported Co_3_O_4_ microtrepangs as OER catalyst but N‐doped carbons as ORR catalyst to assemble Zn–air battery.^194^ Zn–air battery exhibited a long cycle life of 200 cycles, and no significant changes in round‐trip overpotential after charge/discharge at 10 mA cm^−2^ for 800 h. Following nanomaterials engineering in doping and hybridizing with other functional nanocarbons, ORR activity of Co_3_O_4_ can also be enhanced recently. One typical example was 3D ordered mesoporous Co_3_O_4_ (3DOM Co_3_O_4_).^195^ Benefiting from the high active surface area and stable structure, this 3DOM Co_3_O_4_ was proved to be a promising candidate as bifunctional oxygen electrocatalyst (**Figure** **8**a–c). Combining with other active metal oxides such as MnO_2_ and/or coupling with carbon materials, the composites further demonstrated the enhanced catalytic activity for oxygen electrochemistry.^196^ For instance, Xu et al. used Co_3_O_4_/MnO_2_‐CNTs^197^ and La_2_O_3_/Co_3_O_4_/MnO_2_‐CNTs to assemble Zn–air batteries (Figure 8d–f).^198^ The voltage gap of charge/discharge increased only 0.1 V after 543 charge/discharge cycles at 10 mA cm^−2^ for the rechargeable Zn–air battery using Co_3_O_4_/MnO_2_‐CNTs catalysts.^197^ Other Co‐based bifunctional electrocatalysts even coupled with nanocarbons have also been investigated in Zn–air batteries, such as CoMn_2_O_4_/N‐rGO,^199^ MnCo mixed oxide,^200^ Co(II)_1−_ *_x_*Co(0)*_x_*_/3_Mn(III)_2_ *_x_*_/3_S nanoparticles supported on B/N‐codoped mesoporous nanocarbon,^201^ Co‐PDA‐C,^202^ NiCo_2_O_4_,^81^ NiCo_2_O_4_/NCNT,^203^ Co‐doped TiO_2_,^204^ and CoS*_x_*@ N,S codoped graphene nanosheets.^205^

**Figure 9:**
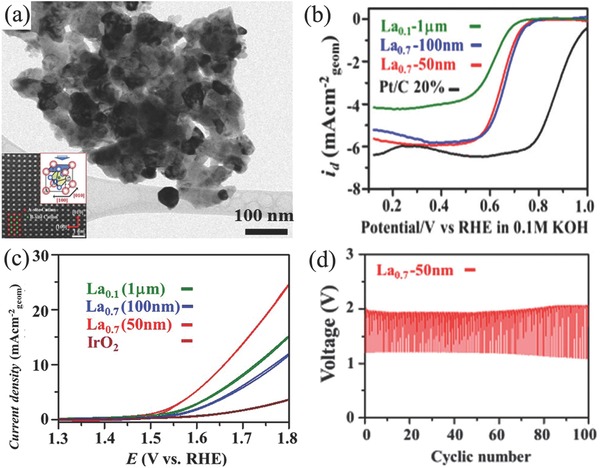
TEM image of La*_x_*(Ba_0.5_Sr_0.5_)_1−_ *_x_*Co_0.8_Fe_0.2_O_3−_ *_δ_* (BSCF) nanoparticles with size of 50 nm (a); ORR (b) and OER (c) catalytic activity for BSCF nanoparticles; the repeated charge and discharge tests of BSCF nanoparticles based Zn–air battery (d). Reproduced with permission.^209^ Copyright 2016, RSC.

Mixed metal oxides with perovskite structure are another kind of promising bifunctional oxygen electrocatalyst and have wide applications in Zn–air batteries.^206^, ^207^ The structure of them are in a general ABO_3_ formulation (A: rare earth or alkali metal ions; B: transition metal ions), in which the B site is usually recognized as catalytic active center. ORR and OER activities of perovskite oxides can be simultaneously improved by filling the surface B antibonding states of e_g_‐orbital close to 1 and can be further increased by enhancing the covalence of B—O bond.^207^, ^208^ For example, Cho and co‐workers optimized the size of La*_x_*(Ba_0.5_Sr_0.5_)_1−_ *_x_*Co_0.8_Fe_0.2_O_3−_ *_δ_* nanoparticles to adjust the catalytic activity for ORR and OER at the same time.^209^ They claimed that the excellent catalytic activity for ORR (onset potential: 0.72 V) and OER (overpotentials at 2 A g^−1^: 1.54 V) can be simultaneously achieved by nanoparticles in the size of ≈50 nm (**Figure** **9**a–c). After 100 charging/discharging cycles, the overpotential difference between charge and discharge of this perovskite assembled Zn–air battery increased only 0.25 V (Figure 9d). In addition, PrBa_0.5_Sr_0.5_Co_2−_ *_x_*Fe*_x_*O_5+_ *_δ_* (*x* = 0, 0.5, 1, 1.5, and 2) mesoporous nanofibers^210^ and La_0.8_Sr_0.2_Co_1−_ *_x_*Mn*_x_*O_3_ (*x* = 0, 0.2, 0.4, 0.6, 0.8, 1) nanostructures^211^ have also been reported. The electrocatalytic activity of these materials can be adjusted by the B‐site metal ratio, and the best performance can be achieved at *x* = 0.5 and 0.6, respectively. Besides, lanthanum based LaMO_3_ perovskite oxide is widely investigated in the area of Zn–air batteries as well, such as doped La_2_NiO_4_,^212^ LaFeO_3_ nanostructures,^213^ and LaCoO_3_ fibers.^190^ When being assembled into rechargeable Zn–air batteries, both of them can exhibit good properties. Moreover, the synergistic effect of La_2_NiO_4_ nanoparticles and carbon nanotubes^214^ or nitrogen‐doped carbon^215^ was found and it can further enhance the performance of Zn–air battery.

**Figure 10:**
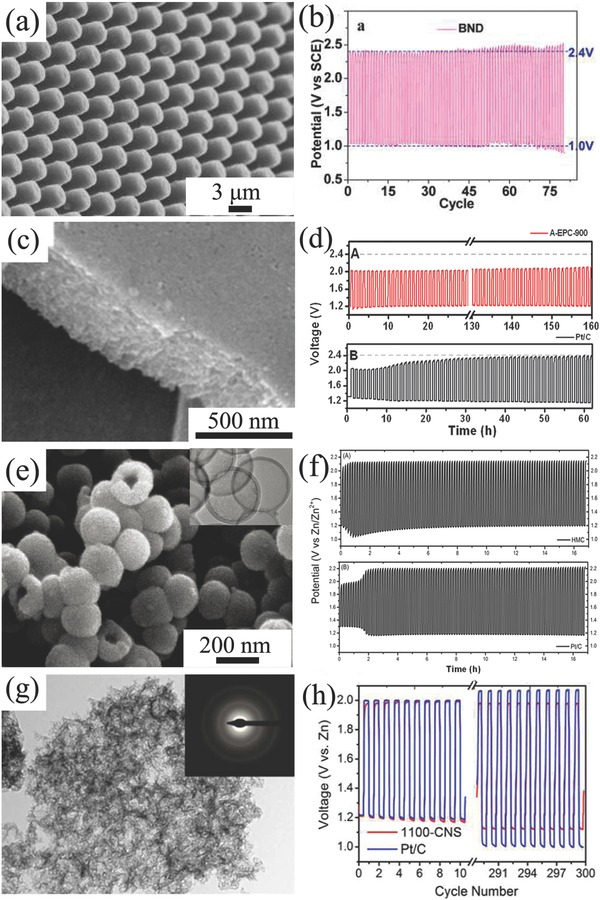
a) SEM image and b) discharge/charge cycling curve of Zn–air battery assembled by B, N‐codoped nanodiamond at 16.0 mA cm^−2^. Reproduced with permission.^223^ Copyright 2013, ACS. c) SEM image and d) discharge/charge cycling results of microporous carbon sheets and Pt/C based Zn–air batteries at 2 mA cm^−2^. Reproduced with permission.^224^ Copyright 2015, RSC. e) SEM and TEM images and f) corresponding discharge/charge cycling curves of hollow mesoporous carbon and Pt/C based Zn–air batteries at 2 mA cm^−2^. Reproduced with permission.^225^ Copyright 2015, RSC. g) TEM image and h) discharge/charge cycling curves of hierarchically porous carbon and Pt/C based Zn–air batteries at 10 mA cm^−2^. Reproduced with permission.^226^ Copyright 2017, RSC.

After the discovery of doped carbon materials in ORR application, nanocarbons were further found to exhibit OER activity individually or simultaneously.^216^, ^217^ Then cheap, highly reserved and safe carbon‐based materials were further investigated as oxygen electrocatalysts in the area of rechargeable Zn–air batteries.^218^, ^219^ Although the main concerns of carbon oxidation and corrosion at high potentials for most of carbon‐based catalysts studied,^147^ graphitic carbons,^220^, ^221^ and diamond carbons still display the great advantages of higher resistance to electrochemical oxidation and corrosion.^222^ For example, Zn–air battery used N,B codoped diamond as bifunctional oxygen electrocatalyst showed a power density of 24.8 mW cm^−2^ and a good stability of 80 cycles at a charge/discharge current density of 16 mA cm^−2^ (**Figure** **10**a,b).^223^ Another sample is microporous carbon sheets synthesized from eggplant, which exhibited a specific capacity of ≈669 mA h g_Zn_^−1^ and was stably charged and discharged for 160 h at 2 mA cm^−2^ (Figure 10c,d).^224^ Both of them demonstrated excellent cycle performance and a long operation time without significant corrosion of carbon electrocatalyst during the charge/discharge process. With further structure optimizing, porous nanocarbons with high surface area such as hollow N‐doped carbon spheres were also employed as the electrocatalyst for Zn–air battery (Figure 10e).^225^ The potential gap of this assembled Zn–air battery increased just 40 mV after charge/discharge at 2 mA cm^−2^ for 5 h (Figure 10f). The similar method was also employed to prepare N,S‐codoped hierarchically porous carbon material as bifunctional oxygen electrocatalyst (Figure 10g,h).^226^

**Figure 11:**
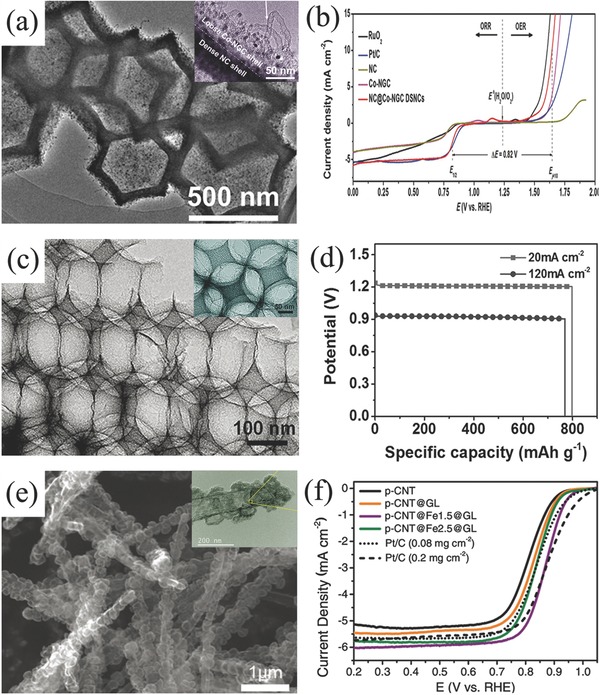
a) TEM image and b) OER and ORR polarization curves of double shelled carbon material derived from ZIF‐67@ZIF‐8. Reproduced with permission.^230^ Copyright 2017, Wiley. SEM image (c) and specific capacity of Zn–air battery employed photonic crystal architecture carbon (d). Reproduced with permission.^232^ Copyright 2017, Wiley. SEM image (e) and ORR performance of Fe–N‐porous carbon nanotubes covered with graphitic layer (f). Reproduced with permission.^233^ Copyright 2017, Wiley.

As containing atomically dispersed metal species and abundant nitrogen/carbon source, metal–organic frameworks (MOFs) is also an important platform material in the area of bifunctional oxygen electrocatalysts and rechargeable Zn–air batteries.^227^ For example, Liu et al. used Zn‐doped ZIF‐67 to acquire bifunctional oxygen catalyst.^228^ The Zn–air battery employed this catalyst can exhibit a high gravimetric energy density (889 Wh g_Zn_^−1^), but the cycling performance was not that well as a stable charge/discharge for 33 h at current density of 7 mA cm^−1^. Zhao et al. directly heated the MC‐BIF‐1S and obtained N,B‐doped carbon material as a bifunctional catalyst for rechargeable Zn–air battery.^229^ This battery showed an excellent cycle performance as charge/discharge for 100 h at current density of 2 mA cm^−1^ without obvious performance loss. Zhao and co‐workers carbonized ZIF composite and obtained double shelled carbon nanocages (**Figure** **11**a).^230^ This novel structure showed a similar ORR (half‐wave potential: 0.79 V) and better OER (*E_j_*_= 10_: 1.64 V) catalytic activity than both ZIF‐8 (ORR half‐wave potential: 0.73 V; OER *E_j_*_= 10_: 1.70 V) and ZIF‐67 (ORR half‐wave potential: 0.8 V; OER *E_j_*_= 10_: 1.68 V) derived carbon materials (Figure 11b). The optimized porous structures for MOF‐derived catalyst also promote the electrochemical properties, such as the template method to create dimensional porous structures.^231^ Chen et al. utilized 3D ordered SiO_2_ microspheres as template and prepared N‐doping porous carbon material with 3D photonic crystal architecture (Figure 11c).^232^ This 3D structure showed a fantastic surface area value of 2546 m^2^ g^−1^, when used to Zn–air battery, this battery exhibited an ultrahigh capacity (770 mA h g_Zn_^−1^) as shown in Figure 11d. Ahn et al. assumed tellurium (Te) nanotube as template and covered it with ZIF‐8 and Fe‐PDA.^233^ After thermolysis and removal of Te, Zn, and Fe, N‐doped porous carbon nanotubes embedded with FeN*_x_*C active sites were formed (Figure 11e). The 1D porous structure of carbon nanotubes and FeN*_x_*C active sites would induce synergistic effects and guaranteed the excellent ORR and OER catalytic activity especially ORR catalytic activity. As shown in Figure 11f, it exhibited a better potential of 0.957 V at *j* = 0.3 mA cm^−2^ and *E*_half_ of 0.867 V than unmodified porous carbon nanotubes. Song et al. grew MOFs on the surface of ZnO microspheres template and prepared hollow Co–N‐doped carbon materials.^234^ Zn–air batteries using this material acquired an excellent open circuit potential of 1.59 V and a high‐power density of 331.0 mW cm^−2^. Besides, rechargeable Zn–air battery assembled by ZIF‐selenized Co_0.85_Se nanocrystal showed a narrow discharge/charge voltage gap (0.8 V) and stable cycling performance (180 h) at current density of 10 mA cm^−2^.^235^ Hybridizing with other functional materials such as graphene,^236^ Mxene,^237^ and metal oxides,^238^ MOFs‐derived nanocomposites further demonstrated better oxygen electrochemical performance.

**Table** **2** summarizes some advanced oxygen electrocatalysts performed in rechargeable Zn–air batteries. According to this table, Co_3_O_4_ based nanomaterial is the most used among those potential bifunctional oxygen electrocatalysts. In addition, most of them exhibit good OER catalytic activity. This phenomenon is also present for mixed metal oxides with perovskite structure. In the opposite, carbon based materials prefer ORR activity rather than OER activity. Moreover, MOFs derived porous materials possess much bigger specific surface area which is beneficial for the three‐phase boundary reaction in Zn–air batteries. Thus, the suitable electrocatalysts or composites can be selected according to different requirements of batteries.

**Table 2:** Summary of bifunctional catalysts performed in rechargeable Zn–air batteries

Catalysta)	ORR activity	Test condition	OER activity	Battery performancea)	Electrolyte	Ref.
Pt/C (ORR); IrO_2_@Ti (OER)	–	0.1 m H_3_PO_4_ + 1 m LiH_2_PO_4_	Tafel slope: 121.8 mV dec^−1^	Open‐circuit voltage: 2.1 V; cycling ability: 50 cycles (200 h)	LiOH‐H_3_PO_4_ + 1 m LiH_2_PO_4_	217
Ni_3_FeN‐ordered Fe_3_Pt intermetallic nanoalloy	*n* = 4	0.1 m KOH	Overpotential 0.365 V at 10 mA cm^−2^	Cycling ability: 480 cycles (40 h) @ 10 mA cm^−2^	6 m KOH	173
Atomically Pt‐CoO	Tafel slope: 43 mV dec^−1^	0.1 m KOH	–	Cycling ability: 30 cycles (charge @ 10 mA cm^−2^; discharge@ 5 mA cm^−2^)	6 m KOH	185
Co_3_O_4_	Onset potential: −0.197 V vs SCE; Tafel slope: 72 mV dec^−1^	0.1 m KOH	Tafel slope: 58 mV dec^−1^	Power density: ≈73 mW cm^−2^; cycling ability: 200 cycles@10 mA cm^−2^	6 m KOH + 0.2 m Zn(Ac)_2_	195
Co_3_O_4_	Onset potential: 0.91 V	0.1 m KOH	–	Cycling ability: 400 cycles @ 5 mA cm^−2^	6 m KOH	191
Co_3_O_4_ NW array/steel mesh	–	–	–	Cycling ability: 600 h @ 20 mA	6 m KOH	219
LaCoO_3_	Onset potential: −0.145 V; *n* = 3.43	0.5 m KOH	Onset potential: 0.693 V	–	–	190
La_2_NiO_4_	Onset potential: 0.91 V vs RHE; *n* = 3.6–3.9	0.1 m KOH	–	Voltage gap: ≈1.51 V; cycling ability: 20 cycles (50 min) @ ≈25 mA cm^−2^, polarization increased 0.4 V at the end	6 m KOH	210
Mn‐Co mixed oxide	Onset potential: 0.076 V; *n* = 3.34	0.1 m KOH	Overpotential: 0.246 V	Open circuit potential: 1.53 V; cycling ability: 60 cycles (30 h) @ 5 mA cm^−2^	6 m KOH	200
MnO_2_/Co_3_O_4_	Onset potential: 1.05 V; Tafel slope: 58 mV dec^−1^	0.1 m KOH	Tafel slope: 34 mV dec^−1^	Cycling ability: 60 cycles (7 h) @ 15 mA cm^−2^, polarization increased ≈0.3 V at the end	6 m KOH	192
CoO nanoclusters and high‐index face Mn_3_O_4_ nano‐octahedrons	*n* = 3.8	0.1 m KOH	Overpotential: 378 mV	Cycling ability: 250 cycles @5 mA cm^−2^	6 m KOH	193
NiCo_2_O_4_	Onset potential: 0.93 V vs RHE	0.1 m KOH	Potential at 10 mA cm^−2^: 1.62 V; Tafel slope: 87 mV dec^−1^	Open circuit potential: 1.45 V; Discharge capacity: 580 mA h g^−1^; cycling ability: 50 cycles (1000 min) @ 20 mA cm^−2^	6 m KOH	81
NiFeO@MnO*_x_* core–shell structures	*n* = 3.8; onset potential; 0.88 V	0.1 m KOH	Tafel slope: 37–46 mV dec^−1^	Cycling ability: 100 cycles @ 2 mA cm^−2^	6 m KOH	176
Pb_2_Ru_2_O_6.5_	*n* = 4; onset potential: 0.89 V vs RHE	0.1 m KOH	Tafel slope: 114.2 mV dec^−1^	Cycling ability: 200 cycles (2000 min)@ 50 mA cm^−2^	6 m KOH	175
Nanocrystalline yttrium ruthenate pyrochlore	Onset potential: 0.85 V vs RHE; *n* = 3.8	0.1 m KOH	Overpotential: 1.45 V; Tafel slope: 112.4 mV dec^−1^	Cycling ability: 200 cycles (2000 min) @10 mA cm^−2^	6 m KOH	174
Co‐doped TiO_2_	Onset potential: −0.14 V vs Hg/HgO; *n* = 3.55–3.85	0.1 m KOH	Overpotential: 0.347 V vs Hg/HgO @ 10 mA cm^−2^	Peak power density: 136 mW cm^−2^; cycling ability: 37 cycles (750 h) @ 20 mA cm^−2^	6 m KOH	204
Co_3_FeS_1.5_(OH)_6_	Half wave‐potential: 0.721 V vs RHE; Tafel slope: 79 mV dec^−1^	0.1 m KOH	1.588 V vs RHE @ 10 mA cm^−2^	Specific capacity: 898 mA h g^−1^; Cycling ability: 108 cycles @ 2 mA cm^−2^	6 m KOH	138
NiMn LDH	–	0.1 m KOH	Overpotential: 0.35 V; Tafel slope: 40 mV dec^−1^	Cycling ability: 55 h @10 mA cm^−2^	6 m KOH + 0.2 m Zn(Ac)_2_	49
Co_5_AlS_1.5_(OH)_6_	–	–	Tafel slope: 79 mV dec^−1^	Specific capacity: 898 mA h g^−1^; cycling ability: 108 cycles @2 mA cm^−2^	6 m KOH + 0.2 m Zn(Ac)_2_	138
NiO/CoN porous nanowires	*n* = 3.97	0.1 m KOH	Δ*E* = 0.85 V	Open‐circuit: 1.46 V; power density; 79 mW cm^−2^ @ 200 mA cm^−2^; cycling ability: 50 cycles (500 min) @ 50 mA cm^−2^	6 m KOH	182
Ni_3_FeN	*n* = 3.79–3.87	0.1 m KOH	Tafel slope: 70 mV dec^−1^	Cycling ability: 310 cycles (170 h)	6 m KOH + 0.2 m ZnCl_2_	52
Microporous carbon sheets	Onset potential: ≈69 mV vs Ag/AgCl; *n* ≈ 4	0.1 m KOH	Less positive onset potential than Pt/C	Discharge voltage: 1.23 V; cycling ability: 160 cycles (160 h) @ 2 mA cm^−2^	6 m KOH	224
Hollow N‐doped mesoporous carbon spheres	Onset potential: −0.055 V vs Hg/HgO; *n* ≈ 4	0.1 m KOH	Onset potential: 0.365 V vs Hg/HgO	Cycling ability: 30 cycles (5 h) @ 2 mA cm^−2^, polarization increased 0.04 V at the end	6 m KOH	225
B‐N codoped porous carbon	Onset potential: 0.894 V; *n* = 3.6	0.1 m KOH	Onset potential: 1.38 V; Tafel slope: 201 mV dec^−1^	Discharging voltage: 1.14 V; 52%; cycling ability: 600 cycles (100 h) @ 2 mA cm^−2^	6 m KOH	229
N,B‐doped diamond	Onset potential: −0.05 V vs SCE; *n* = 3.96	0.1 m KOH	–	Power density: 24.8 mW cm^−2^; cycling ability: 80 cycles @ 16 mA cm^−2^	6 m KOH	219
N,P‐doped carbon foam	Onset potential: 0.94 V vs RHE; half‐wave potential: 0.85 V vs RHE; *n* = 3.85	0.1 m KOH	–	Open‐circuit potential: 1.48 V; energy density: 835 Wh kg_Zn_^−1^; power density: 55 mW cm^−2^; cycling ability: 180 cycles @ 2 mA cm^−2^	6 m KOH	188
N,S‐doped porous carbon	Onset potential: 0.99 V; half‐wave potential: 0.85 V; Tafel slope: 58 mV dec^−1^	0.1 m KOH	Tafel slope: 292 mV dec^−1^.	Power density: 151 mW cm^−2^; cycling ability: 55 h @ 10 mA cm^−2^	6 m KOH + 0.2 m Zn(Ac)_2_	226
Single‐walled NCNTs/Ag	–	–	–	Open‐circuit voltages: ≈1.2 V; specific energy density: 300 Wh kg^−1^; specific capacity: 515 mA h g^−1^	6 m KOH	160
Co‐PDA‐C	Half‐wave: 767 mV vs RHE; *n* = 3.5–3.8	0.1 m KOH	1.601 V (2 mA cm^−2^)	Cycling ability: 500 cycles @ 2 mA cm^−2^, polarization increased 0.23 V at the end	6 m KOH	202
Co‐PDA‐N codoped carbon	*n* = 3.5–3.8	0.1 m KOH	Potential of 1.601 V is 45 mV less positive than that of Pt/C at 2 mA cm^−2^	Cycling ability: 500 cycles (500 h)@ 2 mA cm^−2^	6 m KOH	202
MO‐Co@N‐carbon	*n* = 3.87	0.1 m KOH	Tafel slope: 77 mV dec^−1^	Cycling ability: 385 cycles (3850 min) @ 10 mA cm^−2^	6 m KOH + ZnCl_2_	183
C‐Fe‐UFR	Onset potential: 1.01 V; half‐wave: 0.86 V	0.1 m KOH	Tafel slope: 160 mV dec^−1^	Specific capacities: 467 mA h·g_Zn_^−1^ @ 10 mA·cm^−2^; cycling ability: 100 cycles (2000) @ 10 mA cm^−2^	6 m KOH + 0.2 m Zn(Ac)_2_	269
Ni_3_Fe/N‐carbon sheets	Onset potential: 0.90 V	0.1 m KOH	Tafel slope: 77 mV dec^−1^	Cycling ability: 105 cycles	6 m KOH + 0.2 m ZnCl_2_	184
Fe/Fe_2_O_3_@Fe‐N‐C	Onset potential: −0.04 V	0.1 m KOH	Tafel slopes: 77.5 mV dec^−1^	Open circuit voltage: 1.47 V vs Ag/AgCl; power density: 193 mW cm^−2^ @ 220 mA cm^−2^	6 m KOH	93
CoO/NCNT (ORR); NiFe‐LDH/CNT (OER)	Onset potential ≈20 mV negative to that of Pt/C	6 m KOH	At 50 mA cm^−2^, ≈20 mV negative than Ir/C benchmarked	Power density: 256 mW cm^−2^; cycling ability: 20 cycles (200 h) @ 20 mA cm^−2^	6 m KOH	142
RuO_2_‐ordered mesoporous carbon nanofiber arrays	Half‐wave potential: 0.8 V	0.1 m KOH	Tafel slope smaller than Pt/C	Cycling ability: 100 cycles (2000 min) @ 4 mA cm^−2^	6 m KOH	180
N‐doped carbon (ORR); Co_3_O_4_ @Ni(OER)	Half‐wave potential: 0.82 V vs RHE; *n* ≈ 4	0.1 m KOH	Tafel slope: 49 mV dec^−1^	Voltaic efficiency: 64.5%; cycling ability: 200 cycles (800 h)@10 mA cm^−2^	6 m KOH	194
Co_3_O_4_/carbon nanofibers	Half‐wave: −0.188 V vs Ag/AgCl; *n* = 4	0.1 m KOH	Potential at 2 mA cm^−2^: 0.64 V vs Ag/AgCl; Tafel slope: 23 mV per decade	Power density: 125 mW cm^−2^; cycling ability: 135 cycles (135 h) @ 1 mA cm^−2^, polarization increased ≈0.08 V at the end	6 m KOH	163
CoO*_x_* nanoarrays with porous N‐carbon	–	0.1 m KOH	Δ *E* = 0.78 V	Cycling ability: 110 cycles	6 m KOH + 0.2 m Zn(Ac)_2_	50
Co_3_O_4_/MnO_2_‐CNTs	Onset potential: 0.958 V; Tafel slope: 113 mV dec^−1^	0.1 m KOH	Tafel slope: 61.5 mV dec^−1^	Power density: 450 mW cm^−2^	6 m KOH	197
NCNT/Co*_x_*Mn_1−_ *_x_*O	Onset potential: 0.96 V; *n* = 3.8	1.0 m KOH	Tafel slope: 40 mV dec^−1^	Gravimetric energy density: 695 W h kg_Zn_^−1^; cycling ability: 12 h @ 7 mA cm^−2^	6 m KOH	64
CoMn_2_O_4_/N‐rGO	Onset potentials: 0.87 V vs RHE	0.1 m KOH	OER of 10 mA cm^−2^ at 1.66 V	Charge/discharge voltage gap: 0.70 V; cycling ability: 100 cycles (500 min) @20 mA cm^−2^, polarization increased ≈0.2 V at the end	6 m KOH	199
MnCo_3_O_4_/N‐carbon nanofiber arrays	Onset potential: 0.9 V vs RHE	0.1 m KOH	Better than RuO_2_	Cycling ability: 100 cycles (2000 min) @ 10 mA cm^−2^	6 m KOH + 0.2 m ZnCl_2_	51
NiCo_2_O_4_/NCNT	Onset potential: 0.934 V; Tafel slope: 155 mV dec^−1^	0.1 m KOH	OER current density at 1.7 V is 16 mA cm^−2^	Power density: 320 mW cm^−2^; voltage polarization: ≈0.75 V @ 10 mA cm^−2^; cycling ability: 60 h @ 10 mA cm^−2^	6 m KOH	203
MnO_2_‐NCNT	–	–	–	Cycling ability: 50 cycles (250 min) @ 8 mA cm^−2^, polarization increased ≈0.4 V at the end	6 m KOH	218
La_2_O_3_/Co_3_O_4_/MnO_2_‐CNTs	Onset potential: 0.93 V; *n* = 3.9	0.1 m KOH	Onset potential: 1.42 V	Power density: 295 mW cm^−2^; cycling ability: 543 cycles (90.5 h) @ 10 mA cm^−2^, polarization increased 0.1 V at the end	6 m KOH	198
LaNiO_3_/NCNT	Half‐wave potential: similar to commercial Pt/C.	–	–	Cycling ability: 75 cycles (375 min) @ 17.6 mA cm^−2^, polarization increased 0.1–0.2 V at the end	6 m KOH	220
Atomically disperses Fe‐N*_x_*/N, S‐hierarchical carbon layers	–	0.1 m KOH	Tafel slope: 82 mV dec^−1^	Power density: 102.7 mWcm^−2^;cycling ability: 100 cycles @ 5 mA cm^−2^	6 m KOH	221
Ni‐Fe nitride/N‐ graphene	Onset potential: 0.9 V vs RHE	0.1 m KOH	Overpotential: 400 mV at 10 mA cm^−2^	Cycling ability: 180 cycles (30 h) @ 10 mA cm^−2^	6 m KOH	152
Ni_3_FeN/Co, N‐CNF	Half‐wave potential: 0.81 V; Tafel slope: 52 mV dec^−1^	0.1 m KOH	Tafel slope: 51 mV dec^−1^	Cycling ability: 540 h @ 6 mA cm^−2^; 136 h @ 50 mA cm^−2^	6 m KOH + 0.2 m Zn(Ac)_2_	151
CoS*_x_*@ N,S codoped graphene nanosheets	Onset potential: −0.174 V); *n* = 3.2	0.1 m KOH	Onset potential: 0.674 V	Cycling ability: 50 cycles @ 1.25 mA cm^−2^	6 m KOH	205
Co(II)_1–_ *_x_*Co(0)*_x_*_/3_Mn(III)_2_ *_x_*_/3_S/B/N‐Codoped mesoporous carbon	*n* ≈ 4	0.1 m KOH	Onset potential: 1.49 V vs RHE (1 m KOH); Tafel slope: 50 mV dec^−1^	Power density: 250 mW cm^−2^; specific capacity: ≈550 mA h g^−1^; charge/discharge voltage gap: ≈0.72 V @ 20 mA cm^−2^	6 m KOH	201
NiCo_2_S_4_/N‐CNTs	*n* = 3.8	0.1 m KOH	∆*E* = 0.80 V	Cycling ability: 150 cycles @10 mA cm^−2^	6 m KOH	141

^a)^ The detail test condition refers to the primary references.

## 5 Flexible Zn–Air Batteries

Except for the development of power battery, high market demands in consumer electronics especially portable and wearable devices also motive the new evolution of Zn–air batteries with some advanced features such as lightweight and shape conformability in the small unit.^239^ Therefore, developing suitable power supply system becomes imperative, Zn–air battery with flexibility and stretchability is then highly desirable.^240^ To successfully achieve this concept, flexibility of each component (cathode, anode, separator, and electrolyte) is the vital matter to obtain its stable battery performance during the repetitive external strain force.^241^ A summary of air electrodes and electrolytes employed in flexible Zn–air batteries could be seen in **Table** **3**.

**Table 3:** Summary of flexible Zn–air batteries

Anodea)	Electrolyte	Air electrode		Cell structure	Battery performancea)	Ref.
		Catalyst	Current collector			
Primary batteries						
Spiral zinc plate	Gelatin‐0.1 m KOH	Fe/N/C	–	Cable‐type	Power densities: 217 mW cm^−2^	268
Zinc powder	Polypropylene separator‐9 m KOH	Silver ink	Nano‐silver conductive ink on PP membrane	Sandwich structure	Energy density: 682 Wh kg^−1^	181
Zinc foil	6 m KOH	SWCNT	SWCNT	Sandwich structure	Discharge capacity: 375 mA h g^−1^ @ 0.25 mA	264
Recharge batteries						
Zn spring	PVA‐PEO‐KOH	RuO_2_/CNT sheet	CNT sheet	Fiber‐shaped	Energy density: 6 Ah L^−1^; power density: 5.7 Wh L^−1^; cycling ability: 30 cycles @ 1A g^−1^	164
Zn plate	PVA‐KOH gel	CuCo_2_O_4_/N‐CNTs	Carbon cloth and nickel foam	Cable‐type	Open‐circuit potential: 1.24 V; power density: 1.86 W g^−1^; cycling ability: 27 cycles (13.5 h) @ 0.5 A g^−1^	34
Spring Zn belt	PVA‐KOH	Co_4_N/Co‐N‐C	Carbon fiber network	Cable‐type	Open‐circuit voltage: 1.346 V; cycling ability: 36 cycles (12 h) @ 0.5 mA cm^−2^	269
Zn powder, carbon and polymer binder	Cellulose nanofibers	Co_3_O_4_ nanoparticles	Carbon cloth	Sandwich structure	Power density: 2362 mW g^−1^ @ 4650 mA g^−1^; cycling ability: 35 cycles (35 h) @ 250 mA g^−1^	255
Zinc film	Laminate nanocellulose/GO/quaternary ammonium groups	Co_3_O_4_	Carbon cloth	Sandwich structure	Open circuit voltage: 1.4 V; Power density: 44.1 mW cm^−2^; cycling ability: 30 cycles (10 h) @ 1 mA cm^−2^	256
Zn on PET loading with Cu film	PVA‐KOH	Ultrathin Co_3_O_4_ layer	Carbon fibers	Sandwich structure	Cycling ability: 30 cycles (10 h) @ 2 mA cm^−2^	253
Zn foil	PVA‐KOH gel polymer	Carbon nanofiber films	Carbon nanofiber films	Sandwich structure	Open‐circuit voltage: 1.48 V; peak power density: 185 mW cm^−2^; energy density: 776 Wh kg^−1^; cycling ability: 500 cycles @ 10 mA cm^−2^ (voltage gap increased ≈ 0.13 V)	167
Zn plate	PVA‐KOH gel	FeCo/N‐graphitic carbon nanotubes	Carbon cloth	Sandwich structure	Open‐circuit potential: 1.25 V; power density: 97.8 mW cm^−2^; cycling ability: 72 cycles (12 h) @ 100 mA cm^−2^	254
Zn foil	Solid electrolyte	MnO*_x_*‐ Graphene coated carbon cloth	Ti mesh	Sandwich structure	Open‐circuit potential: 1.427 V; cycling ability: 145 cycles	162
Zn foil	PVA‐KOH	Co_3_O_4_/N‐CNT aerogel	N‐CNT aerogel	Sandwich structure	Open circuit voltage: 1.3 V; cycling ability: 20 cycles (20 h) @ 2 mA cm^−2^	240
Zn film	Cellulose film	Co_3_O_4_/NCNT	Stainless‐steel mesh	Sandwich structure	Energy density: 847.6 Wh kg^−1^; cycling ability: 600 h @ 25 mA cm^−2^	245
Zn film	PVA‐gelled electrolyte	LaNiO_3_/NCNT	Carbon cloth	Sandwich structure	Volumetric energy density: 2905 Wh L^−1^; gravimetric energy density: 581 Wh kg^−1^ @ 125 A L^−1^ (25 A kg^−1^); cycling ability: 120 cycles (40 h) @ 250 A L^−1^ (50 A kg^−1^)	270

^a)^ The detail test condition refers to the primary references.

### 5.1 Flexible Electrode

In the flexible Zn–air batteries, Zn anodes used are often in the foil, mesh, plate form, even a gelled mixture of zinc powders with some additives coating in a flexible current collector. While air electrodes should break through the disadvantages of tradition air electrodes such as heavy, rigid, and bulk configuration, thus flexible devices could be achieved, further excellent catalytic activity but yet good mechanical properties of air electrodes should be featured upon bending, folding, or twisting during the fabrication process.^242^

**Figure 12:**
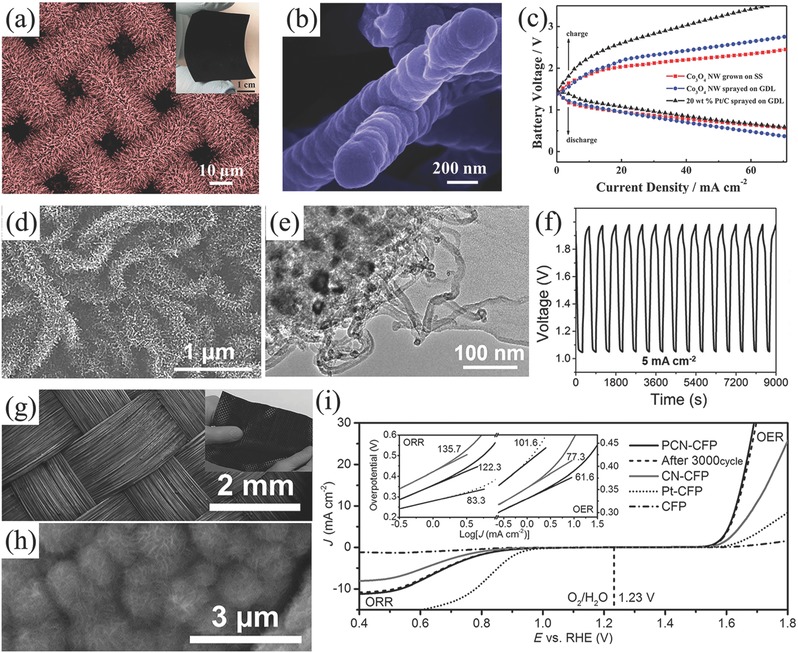
SEM images of SS mesh current collector coated with Co_3_O_4_ NW array (a,b); galvanodynamic discharge/charge curves obtained based on the Co_3_O_4_ NWs grown on SS mesh (c). Reproduced with permission.^243^ Copyright 2014, Wiley. SEM (d) and TEM (e) images of 3D NCNT arrays; galvanostatic discharge–charge curve of the flexible Zn–air battery utilized the 3D NCNT arrays (f). Reproduced with permission.^246^ Copyright 2017, Elsevier. SEM images of PCN‐CFP (g,h); LSV curve of PCN‐CFP at scan rate of 0.5 mV s^−1^ (i). Reproduced with permission.^242^ Copyright 2015, Wiley.

The most straightforward and effective method is to use flexible current collector, as the gas diffusion and catalytic layer can be grown, functionalized, anchored, and embedded onto the current collector to form a flexible battery. For example, Chen and co‐workers selected stainless steel mesh as current collector and directly grew Co_3_O_4_ nanowire array as flexible air electrode (**Figure** **12**a–c).^243^ This approach dramatically simplifies the design and fabrication procedure of flexible air electrode. Meanwhile, electrochemical activity and stability were also enhanced due to the nonconductive ancillary binding material and the reduced resistance due to the direct growth of catalyst on mesh collector. More importantly, the good mechanical strength of SS mesh ensures an excellent bending capability for battery device. Finally, the electrochemical performance of this advanced electrode is demonstrated to be better than Co_3_O_4_ nanowires and Pt/C sprayed on traditional gas diffusion layer based on carbon materials. The chemical coupling of Co_3_O_4_ nanostructures with graphene or mildly oxidized carbon nanotubes can exhibit much higher oxygen electroactivity than free Co_3_O_4_ nanostructures alone.^244^ Chen and co‐workers developed their carbon‐free air electrode, still choosing a flexible stainless‐steel mesh as current collector, but growing 2D mesoporous Co_3_O_4_ nanopetals in 1D N‐doped CNT as the catalyst layer.^245^ The intimate interfacial contact and interaction between Co_3_O_4_ nanopetals and conductive NCNT might provide opportunities to reduce the interface resistance and facilitate charge transfer, while the porous architecture nature of air cathode contributed to the efficient oxygen diffusion. By employing this air cathode and a free‐standing zinc film anode, the produced solid‐state Zn–air battery achieved a high energy density (847.6 Wh kg^−1^) and an outstanding cycling stability (600 h at 25 mA cm^−2^). Li et al. annealed Ni foam decorated with ZIF‐67 to prepare 3D NCNT arrays as air electrode (Figure 12d,e). Even under serious bending stress, flexible Zn–air battery assembled by this air cathode can yield a stable discharge potential of 1.02 V and charge potential of 1.98 V at 5 mA cm^−2^ (Figure 12f).^246^

In view of high tensile strength and good conductivity of graphitic nanocarbon‐based materials such as CNT and graphene, flexible air electrodes can be realized by these carbon‐based current collectors.^247^ In addition, advantages of large availability and light weight for carbon materials could also reduce the weight of whole cell, resulting in a higher gravimetric capacity, which is the most imperative requirement for the portable/wearable electronics.^248^ Qiao and co‐workers reported the fabrication of flexible and reversible oxygen electrode of carbon‐fiber paper covered by phosphorus‐doped graphitic carbon nitrides (Figure 12g–i).^242^ Due to the robust porous 3D network with high conductivity, carbon‐fiber paper presented an excellent flexible current collector. Similar to carbon fibers current collector, other flexible carbon‐based candidates are also employed to replace the conventional metal current collectors.^249^

### 5.2 Electrolyte

As mentioned above, oxygen electrochemical reaction happened at the interface/surface of triple phase zone, thus the mutual contraction of electrolyte with oxygen dissolved on the surface of catalysts determines the efficiency of reaction.^162^ The good contact of electrolyte and catalysts is closely related to the wettability of air cathode. Except the modification/functionalization of catalysts and air electrodes, the properties of electrolytes can also determine the interaction of air electrodes and the diffusion of reaction materials and products.^250^ For flexible Zn–air batteries, liquid electrolytes may suffer from leakage problem during the repeated mechanical deformations, thus non‐liquid electrolytes are more favorable to serve as a species transport medium in Zn–air battery.^28^ Moreover, the advantages of polymer electrolytes such as film‐forming characteristics, high water solubility, sufficient mechanical strength, and conductivity offer desirable both physical and chemical properties. There are three kinds of polymer electrolytes often used in Zn–air batteries including gel polymer, solid polymer and composite polymer electrolytes.

Gel polymer itself is not an electrolyte but formed by incorporating liquid plasticizer and/or ionically conducting electrolyte solutions containing suitable solvents (KOH solution) and compounds into a polymer matrix.^251^ The high contains of electrolyte solution and diffusive transport property like liquid guarantee a better ambient ionic conductivity than their corresponding solid counterparts.^252^ However, the poor mechanical strength and high viscosity would probably cause an internal short circuit.^253^ In order to overcome this shortcoming, the gel electrolyte often cooperates with cross‐linkers to obtain a solid polymer electrolyte and thus enhance the mechanical properties.^254^ For example, Chen and co‐workers reported the enhanced electrolyte stability by self‐crosslinking among the dimethyloctadecyl [3‐(trimethoxysilyl) propyl] ammonium chloride functionalized cellulose nanofibers.^255^ Besides, they also developed a laminated cross‐linked nanocellulose/graphene oxide electrolyte for flexible rechargeable Zn–air battery.^256^

Contrary to gel polymer, solid polymer electrolyte has good mechanical strength and robustness, they were introduced in Zn–air batteries in 1990s.^257^ It is a liquid‐free system and composed by only salts and polar polymer matrix.^258^ Due to its solid nature, the problem of solvent evaporation and battery leakage could be alleviated even eliminated.^259^ And the low convection property would be beneficial for alleviating the electrode corrosion and increasing the battery life. Nevertheless, there is still much work to be done in the future excellent flexible Zn–air batteries due to the current relatively poor contact with air cathode and low ion conductivity.^260^ Composite polymer electrolytes are formed by adding inorganic materials or ionic liquids into the gel polymer electrolyte to obtain an enhanced conductivity and mechanical performance of gel electrolyte.^261^ In practice, the simplest and most used polymer electrolyte for Zn–air batteries is the mixture of KOH solutions and polymer hosts such as polyethylene oxide (PEO), poly vinyl alcohol (PVA), and poly acrylic acid (PAA).^262^ Actually, the single polymers cannot satisfy both needs of the mechanical and ion conductivity acquired simultaneously, the cooperation of both even triple hosts is often required to improve their ionic mobility.^263^

In the present flexible Zn–air batteries, all the employed electrolytes are gel polymer in consideration of conductivity and simple synthesis, although most of them are claimed to be all‐solid.^264^ As mentioned, this water enriched electrolyte is faced with poor mechanical property and water evaporation.^265^ What's more, even employing gel polymer, flexible battery is inclined to display a relatively poor electrochemical performance compared to liquid batteries because of the high ionic resistance of polymer electrolyte.^266^ Thus, real highly conducive and all‐solid‐state electrolytes are vital obligation and many efforts are being devoted to their discovery. We believe the real effective all‐solid flexible Zn–air batteries will be grasped in the nearby future.

### 5.3 Configurations of a Flexible Zn–Air Battery

**Figure 13:**
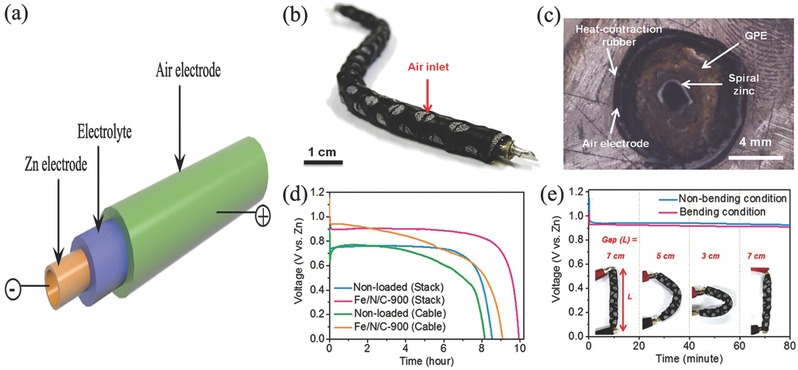
a) Schematic representation, b) photograph, c) cross‐sectional image, d) discharge curves of cable‐type and corresponding stack‐type Zn–air batteries with and without Fe/N/C electrocatalysts, respectively, and e) discharge curves of cable‐type Zn–air battery under bending strain every 20 min at discharge current density of 0.1 mA cm^−2^. Reproduced with permission.^268^ Copyright 2015, Wiley.

There are two configurations for flexible Zn–air batteries. One is the cable‐type configuration, which is widely demonstrated in flexible Li‐ion cells and Li–air batteries (**Figure** **13**a).^267^ The cable‐type Zn–air battery usually has the structure of polymer electrolyte around the surface of central zinc belt, while the outside of polymer electrolyte is coated with flexible air electrode or directly with electrocatalysts. Cho and co‐workers first introduced the cable design into primary Zn–air battery.^268^ This battery was employed a spiral zinc as anode, a gel polymer based on gelatin as electrolyte and air electrode with catalyst made by annealing the mixture of iron acetylacetonate, silk fibroin and ketjenblack (Figure 13b,c). Bending tests exhibited no difference during the discharge process at 0.1 mA cm^−2^ under the bending and nonbending conditions. This result illustrated that cable‐type Zn–air batteries have the ability of efficient operation under the external strain. Liu and co‐workers also reported the similar cable battery with the use of novel Cu–Co bimetallic oxide quantum dots decorated NCNTs as electrocatalyst.^34^ The use of NCNTs substrate not only increased the conductivity of catalyst but also acquired a larger surface area, thus the as‐made flexible Zn–air battery exhibited a stable charging and discharging platforms of 1.29 and 0.98 V, respectively, at current density of 1 A g^−1^. Those charging/discharging potentials can still remain constant when the battery was folded or under serious twist. Zhang and co‐workers also reported the fabrication of flexible and rechargeable cable‐type Zn–air battery, where they employed a free‐standing bifunctional cathode composed of strung Co_4_N and intertwined N‐carbon fibers made by directly carbonization of ZIF‐67/polypyrrole nanofibers network on carbon cloth.^269^ Due to the intimate contact of electrocatalysts and carbon cloth, the assembled cable‐type Zn–air battery exhibited an excellent electrochemical performance and mechanical stability. Under bending stress (bent at 30°, 60°, 90°, and 120°), the discharge voltage at a current density of 0.5 mA cm^−2^ (≈1.23 V) and charge‐transfer resistances (≈17 Ω) remained almost unbothered. Even after bending/stretching of 2000 cycles, the discharge curve dropped only ≈13 mV.

**Figure 14:**
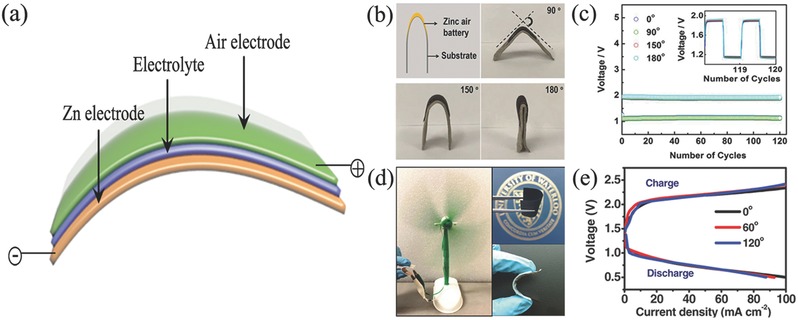
a) Schematic representation of sandwich structure flexible zinc–air battery configuration. b) Optical pictures of flexible zinc–air battery when applied stress to different angles; c) corresponding galvanostatic charge–discharge cycling performance at a current density of 250 A L^−1^ (50 A kg^−1^) under different degree of bending strains. Reproduced with permission.^270^ Copyright 2015, Wiley. d) Optical pictures of flexible zinc–air battery, and e) corresponding charge/discharge polarization curves of battery under bending stress to different angles. Reproduced with permission.^256^ Copyright 2016, Wiley.

Another configuration type for flexible Zn–air batteries is sandwich structure, where anode, electrolyte and air cathode are in plan form and assembled layer by layer (**Figure** **14**a). Fu et al. reported the formation of air electrode through coating Co oxide and perovskite lanthanum nickel oxide/N‐CNT on the flexible carbon cloth substrate. The anode was zinc film, while the electrolyte and separator were gelled PVA membrane (Figure 14b).^270^ This flexible battery exhibited a high volumetric energy density of 2905 Wh L^−1^ and gravimetric energy density of 581 Wh kg^−1^, respectively. The cycling performance was also excellent and could be stable over 120 cycles under a charge/discharge rate of 250 A L^−1^ (50 A kg^−1^). The electrochemical performance was not weakened even at the harsh bending stress (Figure 14c). Zhang et al. assembled a flexible Zn–air battery by employing laminate‐structured nanocellulose/GO based membrane as a solid‐state electrolyte (58.8 mS cm^−1^ at 70 °C), accompanying with zinc film anode and carbon cloth coated Co_3_O_4_/Nafion air electrode (Figure 14d,e).^256^ This device afforded an obvious open‐circuit voltage of ≈1.4 V, and the battery performance remained virtually unchanged at any given bending angles, even at a high current density of 80 mA cm^−2^. Obviously, the intimate contact and strong interaction of robust solid‐state electrolyte layer with anode and air cathode is the main reason for this excellent battery performance under bending conditions. Ma and co‐workers also reported a sandwich structured flexible Zn–air battery which used FeCo/N‐CNT as the bifunctional electrocatalyst, and demonstrated an excellent cycling performance of 144 cycles even at a high current density of 100 mA cm^−2^.^254^

**Figure 15:**
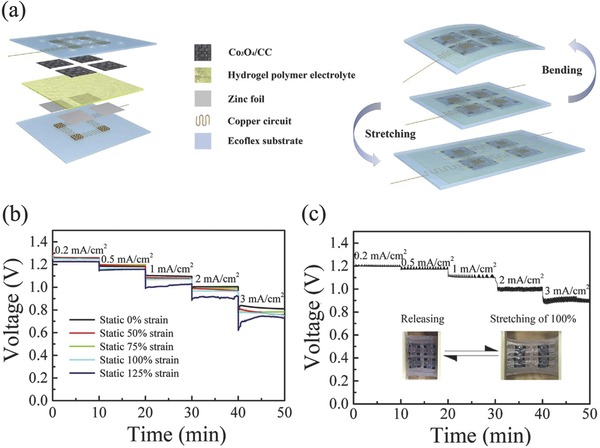
a) Structure illustration of flexible Zn–air battery, b) rate discharge performances of the array under different static strain of 0%, 50%, 75%,100%, and 125% at different current densities, and c) rate discharge performances of the array under dynamically stretching/releasing cycling of a maximum tensile strain of 100% and rate of 2 s per cycle. Reproduced with permission.^272^ Copyright 2017, Elsevier.

To get better mechanical performance, a concept called “break up the whole into parts” strategy was developed for flexible Zn–air battery array recently.^271^ Zhong and co‐workers present a layer‐by‐layer assembly of 2 × 2 electrode arrays.^272^ The air electrode was composed of Co_3_O_4_ nanosheets in situ grown on carbon cloth (**Figure** **15**). The battery array can present significantly stable output voltages even under tensile strains of 100%. But increasing elongation, the discharge voltage plateaus decrease slightly at high current densities. Obviously, besides the electrode and electrolyte, the assembly packing techniques also greatly affect the battery performance through the sufficient contact of each component. In the future, researches related to flexible Zn–air batteries would focus on not only the active electrocatalysts, but also the architectures of air electrode, the conductive electrolytes and the assembly techniques.

## 6 Conclusion and Outlook

Zn–air battery is one of the utmost potential energy technologies because of its high specific density and low cost, which would encounter the ever‐increasing energy demands for portable/wearable electronic devices and electric vehicles. Although considerable progresses have been made in recent years, there are still many problems need to be addressed. For power batteries in electric vehicles and power plants, the low practical energy density is the main hindrance, and consumer batteries also have to take into consideration the mechanical performance, safety and stability in flexible and portable devices. But all of these are closely related to air electrodes and their relevant components, including oxygen electrocatalysts, current collectors, and electrolytes. This review highlights their exciting advances and major issues in aspect of respective components. Thus, the intention of this review is to provide valuable understandings and insights for research community and application sectors to speed up innovation especially in air electrodes related parts for high performance Zn–air batteries.

Oxygen electrocatalyst is the key component in air electrode, which determines the configurations, performance, and cost of Zn–air batteries, thus the first emphasis of air electrode should be highly efficient and robust but cheap oxygen electrocatalysts. Noble metal catalysts exhibit extraordinary catalytic activity, but the inevitable degradation at high charge/discharge potentials in oxygen electrochemical reaction induce the poor cycle ability of battery. Meanwhile the disadvantages of cost and scarcity also limit the wide applications. Thus, progressive attention would be paid on the exploration and preparation of new non‐noble metal catalysts with high activity and improved durability, such as nanocarbons, metal oxides/carbides/nitrides, conductive polymers, metal coordination complexes, metal–organic frameworks, and their composites. By nanotechnologies and material engineering, the density of active sites and intrinsic catalytic capability of catalyst composites would be improved by selecting proper components, controlling morphologies and structures, tuning the physicochemical properties including valence, phases and defects, etc., and coupling with conductive supports/substrates. Moreover, rechargeable batteries need bifunctional ORR and OER catalysts relative to the unifunctional ORR in primary ones. Although three‐electrode configuration or the use of mixed ORR and OER electrocatalyst could provide alternative solutions, bifunctional ORR/OER catalysts are still becoming hot topic as they can provide ORR and OER activity simultaneously in the rechargeable batteries with two‐electrode configuration. Contrast to ORR process, OER process involves oxygen generation during the charging process, thus the optimized pore channels are required to quickly release oxygen bubbles formed on the surface of catalysts, otherwise the battery reaction will be gradually shut off with the resultant poor battery efficiency.

Although the ORR, OER, and bifunctional oxygen electrocatalysts have received great improvements and developed to a variety of species in the fuel cells and other metal–air batteries, the types of electrocatalysts are far from the practical application of Zn–air batteries. Benefiting from the development of oxygen catalysts in fuel cells, water splitting and metal–air batteries, but real, and full understandings of oxygen electrochemical behaviors should be further illuminated. This will be the second investigation emphasis on air electrodes, as different kinds of catalysts in both aqueous and nonaqueous electrolytes exhibit different reaction pathway and mechanisms. By means of advanced characterization techniques and combining high‐throughput calculations and simulations, the achieved molecular or even atomic level observation and understanding would engage the innovation of highly efficient oxygen electrocatalysts and air electrodes.

In the aspect of the substrate, that is another important element and plays the role of current collector and mechanical support to the battery devices, thus the porous structure, good mechanical strength and excellent conductivity should be taken into consideration simultaneously. Although metal current collector satisfies these requirements, for example, Ni foam, the price and weight limited their use to wide market. On the opposite, carbon‐based current collectors are cheap and light‐weight, however, it is too fragile and easy to be oxidized especially in the charging process. Thus, combining the advantages of metal and graphitic nanocarbons collectors will be one of the research emphasis on air electrode. Moreover, sufficient contact between catalyst and current collector must be adequate to ensure easy electron transfer across the interfaces. Directly growing electrocatalysts on current collectors and making binder‐free air electrode could highly enhance the contact and thus improve the electrochemical performance. The intention of integrated electrode design avoids the complex fabrication process of air electrode through the traditional “brick and mortar” method and would play more important role in the future large‐scale assembly of Zn–air batteries.

The proper electrolyte is also important to the performance of Zn–air battery. Except for the excellent ionic conductivity and safety issues, the good cooperation with air electrode will promote the contact of electrolyte and electrocatalysts. Future studies should not only focus on the surface modification or functionalization of catalysts and air electrodes to acquire appreciate wettability but also the rational design and construction of surface/interface between electrolytes and air electrodes. All those are significant to the cooperation of electrolytes and air electrodes in the electrochemical reactions, and also effectively avoid the flooding on catalysts layer, ensure a good gas diffusion layer, prevent the leakage and evaporation of electrolytes. In particular, solid electrolytes would be promising for the assembly of Zn–air batteries, especially flexible ones.

Developing highly efficient air electrode is a system engineering, except material science and chemistry needed for the active and robust materials, the continuous innovations in assembly techniques and engineering are also encouraged always to achieve high‐performance Zn–air batteries. However, most studies focused on oxygen electrocatalysts by the thin film‐rotating‐disk electrode (RDE) technique, the evaluation of whole assembled air electrode in the real cell is therefore required to get the real performance of Zn–air battery. Gratifyingly, a peak power density of 450 mW cm^−2^ is achieved in small‐scale laboratory tests.^197^ Moreover, most studies used pulsed current technique on their home‐made setups to investigate the discharge/charge performance and cycling performance, but few investigations on the rate capability and charge/discharge depth. Therefore, it is necessary to build a universally accepted evaluating system and criteria for Zn–air batteries. In addition, taking advantages of advanced operando characterization techniques, the operation of batteries and chemical reactions during discharging/charging would be observed in situ, and the deep understandings would ensure the future promotion of air electrodes related to Zn–air batteries.

Zn–air batteries have many advantages and air electrodes have great improvements in recent years, nevertheless, they are still in the early stages and have an enormous developing spacing to hit the mark. Future tremendous efforts should be focused on the generation of both high power and energy densities accompanying with long‐life through the rational design, preparation and assembly of air electrodes. Except the high performance, safety and economics requirements from power batteries and consumer batteries, Zn–air batteries should also aim to the market demands, especially the portable/flexible electronic devices. Finally, Zn–air battery is a complicated system engineering, except electrode materials, assembly techniques and battery operation management are also significant for the stable and efficient battery system. We hope this review will offer some useful insights to general researchers and advanced experts for future development of air electrodes and Zn–air batteries. No doubt that intensive efforts on air electrodes would bring continuous innovations and accelerate the commercialization of Zn–air batteries, thereby provide more energy technology choices for sustainable modern society.

## Conflict of Interest

The authors declare no conflict of interest.
